# Efficacy and safety of acupuncture for painful diabetic neuropathy: a systematic review and meta-analysis

**DOI:** 10.3389/fneur.2024.1402458

**Published:** 2024-06-05

**Authors:** Jiaming Liu, Yueqi Lin, Yuheng Huang, Qingyi Yang, Xiaojie Li, Yinglan Ye, Bohui Zheng, Wei Song

**Affiliations:** ^1^The Second Clinical Medical School of Guangzhou University of Chinese Medicine, Guangzhou, Guangdong, China; ^2^The Second Clinical College of Guangzhou University of Chinese Medicine, Guangdong Provincial Hospital of Chinese Medicine, Guangzhou, Guangdong, China

**Keywords:** acupuncture, painful diabetic neuropathy, efficacy, meta-analysis, safety

## Abstract

**Background:**

Painful diabetic neuropathy (PDN) is a common chronic neurological complication of diabetes mellitus. Medications are often used to relieve pain, but with significant side effects. Acupuncture is now a component of pragmatic and integrative treatment for PDN. An increasing number of relevant randomized controlled trials have been published in recent years, but a comprehensive meta-analysis has not yet been performed. The aim of this paper is to verify the effectiveness and safety of acupuncture for PDN by meta-analysis and trial sequential analysis (TSA).

**Methods:**

All participants in this study should have had a PDN diagnosis and the trial group was treated with acupuncture. Eight databases, including EMbase, PubMed, Web of science, Cochrane Library, China Biology Medicine disc (CBM), China National Knowledge Infrastructure (CNKI), Wanfang and Chongqing VIP (CQVIP) were retrieved from inception to 5 April 2023. Meta-analysis was conducted utilizing RevMan 5.3 and Stata 15.0. TSA was performed to assess the adequacy of sample size for the outcomes.

**Results:**

A total of 36 studies, comprising 2,739 PDN patients, were included. Among them, 1,393 patients were assigned to the trial group and 1,346 patients were treated in the control group. Outcomes covers the primary indicator Total effective rate (RR = 1.42, 95%CI [1.34, 1.52], *p* < 0.00001), with 21 studies reported, Pain intensity (SMD = −1.27, 95%CI [−1.58, −0.95], p < 0.00001), with 23 studies reported, and other outcomes, including motor nerve conduction velocity (MCV; MD = 3.58, 95%CI [2.77, 4.38], *p* < 0.00001), sensory nerve conduction velocity (SCV; MD = 3.62, 95%CI [2.75, 4.49], *p* < 0.00001), Depression score (SMD = −1.02, 95%CI [1.58, 0.46]), Toronto clinical scoring system (TCSS; MD = −2.41, 95%CI [−3.37, −1.45], *p* < 0.00001), Quality of life (SMD = 1.06, 95%CI [0.66, 1.46]), traditional Chinese medicine (TCM) syndrome score (MD = −4.99, 95%CI [−6.79, −3.18], *p* < 0.00001), suggesting that acupuncture have an ameliorating effect on PDN in various respect. Egger’s test revealed publication bias for four outcomes. TSA showed that as for Total effective rate, Pain Intensity, MCV and SCV, the number of included studies was sufficient to support the conclusions.

**Conclusion:**

Acupuncture demonstrates significant effectiveness in improving PDN outcomes, including Total effective rate, Pain intensity, MCV, SCV, Depression score, TCSS, Quality of life, TCM syndrome score. But the Adverse events rate is no different in trail group and control group. The publication bias presented in Total effective rate, Pain intensity, MCV and SCV can be remedied by Trim and filling method.

**Systematic review registration:**

Prospero, https://www.crd.york.ac.uk/prospero/display_record.php?RecordID=477295.

## 1 Introduction

Painful diabetic neuropathy (PDN) is a prevalent complication of diabetic peripheral neuropathy (DPN), which belongs to the category of paralysis in Chinese medicine and often characterized as a combination of Qi deficiency and blood stasis (1, 2). The International Association for the Study of Pain characterizes neuropathic pain as “pain triggered or resulting from a primary lesion or malfunction in the nervous system” (3). PDN typically presents bilaterally in the lower extremities, affecting the feet and ankles in a glove and stocking distribution pattern. Patients may also experience abnormal sensations like tingling, itching, and numbness (4). The prevalence of PDN in diabetes mellitus patients ranges from 20% to 24% (5), with a noted neuropathic pain incidence of 13.3% (6). PDN may result from various forms of diabetic neuropathy, with distal sensory neuropathy being the most prevalent. PDN can be categorized into acute or chronic subtypes, as well as stimulus-independent or stimulus-evoked manifestations (7).

While medications such as antidepressants, anticonvulsants, opioids, serotonin and norepinephrine reuptake inhibitors can alleviate PDN symptoms, with significant side effects including neurological issues and cardiovascular risks (8). Existing clinical treatments contain cyclic antidepressants (amitriptyline, promethazine and nortriptyline), serotonin and norepinephrine reuptake inhibitors (duloxetine, venlazapine), anticonvulsants (gabapentin, pregabalin), opioids and so on. However, there are various degrees of neurological inhibition, elevated blood pressure, abnormal cardiac arrhythmias, and other side effects associated with the clinical treatments that are currently in use. Spinal cord stimulation is an alternative, though its efficacy remains uncertain (9).

Acupuncture is a traditional Chinese medical practice with a history spanning over three millennia, which has accumulated a substantial amount of clinical and theoretical data (10). It achieves improvement by activating certain areas on the body’s surface, referred to as acupoints (11). Primary stimulation techniques encompass manual acupuncture (MA), electroacupuncture (EA), and transcutaneous acupoint electrical stimulation. The practice of acupuncture is extensively employed to mitigate or heal a range of illnesses, encompassing endocrine and metabolic disorders, mental and behavioral issues, neurological conditions, circulatory system disorders, skin conditions, musculoskeletal and connective tissue diseases, among others (12). In America, over 3 million adults utilize acupuncture primarily for chronic pain management (13). Acupuncture is gaining global recognition as a complementary treatment for various conditions, including PDN. However, despite of its popularity and minimal side effects, acupuncture is not currently recommended for PDN due to a lack of large-scale, high-quality randomized controlled trials (RCTs) (14).

Technique Trial Sequential Analysis (TSA) is employed to enhance traditional meta-analyses by considering the infliction of type-I errors, calculating the diversity-adjusted required information size (DARIS) for statistical analysis and setting benchmarks for statistical relevance and ineffectiveness (15). Type I error, also known as a false positive error, refers to the situation where there is actually no overall difference, the null hypothesis H0 is true, but by conducting a hypothesis test with P ≤ α at the specified level of significance α, the null hypothesis H0 is rejected, leading to the conclusion of a difference, thus resulting in a false positive occurrence. TSA assesses the reliability and accuracy of study results by monitoring cumulative evidence and sample sizes, thus preventing biases in meta-analytical outcomes.

Recently, there has been an increasing number of published RCTs focusing on acupuncture in PDN. However, a comprehensive meta-analysis on a similar topic is yet to be conducted. Therefore, the objective of this study is to conduct a comprehensive meta-analysis on the effectiveness and safety of acupuncture in the treatment of PDN. Additionally, we will employ TSA to assess whether the current the sample size is sufficient for the clinical application.

## 2 Methods

### 2.1 Literature search strategy

Eight databases, including PubMed, EMbase, Cochrane, Web of science, China National Knowledge Infrastructure (CNKI), China Biology Medicine disc (CBM), Wanfang and Chongqing VIP (CQVIP) were searched from the inception date to 5 April 2023. The detailed search strategies were presented in Supplementary Table 1. The registration number of this article is CRD42023477295 (Prospero, https://www.crd.york.ac.uk/prospero/display_record.php?RecordID=477295).

### 2.2 Inclusion criteria

#### 2.2.1 Type of studies

This review specifically focused on randomized controlled trials that examine the effects of acupuncture on PDN. About the blinding method and publication date, there is no restriction.

#### 2.2.2 Type of participants

In order to be included, participants should have been diagnosed as PDN. The diagnostic criteria include clinical symptoms, disease history, imaging and physical examination. There are no restrictions regarding the age, sex, race, nationality, or medical institution of the participants.

#### 2.2.3 Type of interventions and comparison

The control group in the included studies should be treated with sham acupuncture, western medicine, or standard care, while the intervention of trial group should be acupuncture.

#### 2.2.4 Type of outcomes

The primary outcomes that will be assessed in this review include total efficacy rate and pain intensity. Secondary outcomes contain the nerve sensory conduction velocity (SCV), nerve motion conduction velocity (MCV), depression scores, Traditional Chinese Medicine (TCM) syndrome effect, quality of life, and adverse events.

### 2.3 Exclusion criteria

Duplicate publications were excluded to prevent redundancy and maintain the uniqueness of the dataset. Additionally, studies where the intervention measures in the experimental group involved herbal medicine, massage, or similar modalities were not included. Furthermore, any studies in which the control group interventions included acupuncture were also excluded from the analysis. Studies for which detailed full-text data necessary for statistical analysis were inaccessible were not considered. Finally, any studies that were not directly relevant to human populations were also excluded from this meta-analysis. These exclusion criteria were implemented to uphold the quality and validity of the selected studies and to ensure the robustness of the meta-analytical findings.

### 2.4 Screening and data extraction

The literature obtained will be independently screened based on the predetermined criteria by two investigators. Subsequently, investigators will cross-check their results. In cases of disagreement, the team will discuss and seek the opinion of a third party if necessary. The extracted data will include the primary author’s name, publication date, subjects’ age, types of interventions, and research outcomes.

### 2.5 Quality assessment of risk of bias

Two reviewers (LJM and LYQ) independently evaluated the risk of bias for each included study. The Cochrane Manual on Systematic Review of Interventions (version 5.1.0) identifies the areas of bias that arise from random sequence generation, assignment hiding, blinding, selective reporting of study outcomes, completeness of outcome data, and other biases. Each risk area is divided into three levels: “high bias risk,” “low bias risk,” and “undefined bias risk.” Any disputes arising out of this process will be settled by discussing with the Third Reviewer (YQY) for agreement.

### 2.6 Statistical analysis

The data was analyzed with RevMan (version 5.4). Continuous variables (i.e., Pain intensity, MCV, SCV, Depression, TCSS, Quality of life, TCM syndrome score) were measured using standardized mean difference (SMD) measurements. Dichotomous variables (Total effective rate and Adverse events) were measured by risk ratio (RR). The corresponding 95% confidence interval (CI) was calculated. Based on Cochrane Handbook for Systematic Reviews of Interventions (Version 5.1.0), *p* < 0.05 showed statistically striking differences. Cochrane’s Q statistics and I^2^ statistics were used to examine inter-study heterogeneity, *p* < 0.05 indicated statistically significant differences, and I^2^ value ≥ 50% indicated significant heterogeneity. When I^2^ ≥ 50%, the fixed-effect model (anti-variance) was used, and the random effect model (DerSimonian Laird) was used.

### 2.7 Subgroup analysis

We performed subgroup analyses based on (1) type of acupuncture intervention (i.e., EA, MA) and (2) duration of treatment (i.e., <30 days or ≥30 days).

### 2.8 Sensitivity analysis

A sensitivity analysis was carried out to validate the reliability of the heterogeneity test results by excluding studies on a case by case basis.

### 2.9 Assessment of publication bias

The evaluation of publication bias was performed by using funnel plots. For continuous variables, Egger’s test was employed, while Peters’ test was utilized for dichotomous variables. A *p*-value lower than 0.05 was considered to indicate the existence of publication bias.

### 2.10 TSA analysis

TSA is implemented through the TSA 0.9.5.10 beta.^1^ The estimation of RIS is based on the following statistical indicators: probability of type I error (α = 0.05), probability of type II error (β = 0.2). For dichotomous data, event rate in control group (Pc = 3%, derived from Meta-analysis data) and relative risk reduction (RRR = 35%). For continuous data, we calculated difference in mean and variance using empirical assumptions provided by software. The O’Brien-Fleming boundary was used as the test of repeated significance for cumulative data in most randomized controlled trials. Z-value is calculated by dividing the pooled intervention effect by the standard error. The following indicators were ignored due to too little research volume: Traditional Chinese Medicine (TCM) syndrome effect, quality of life, and adverse events.

## 3 Results

### 3.1 Basic information of literature

**Figure 1:**
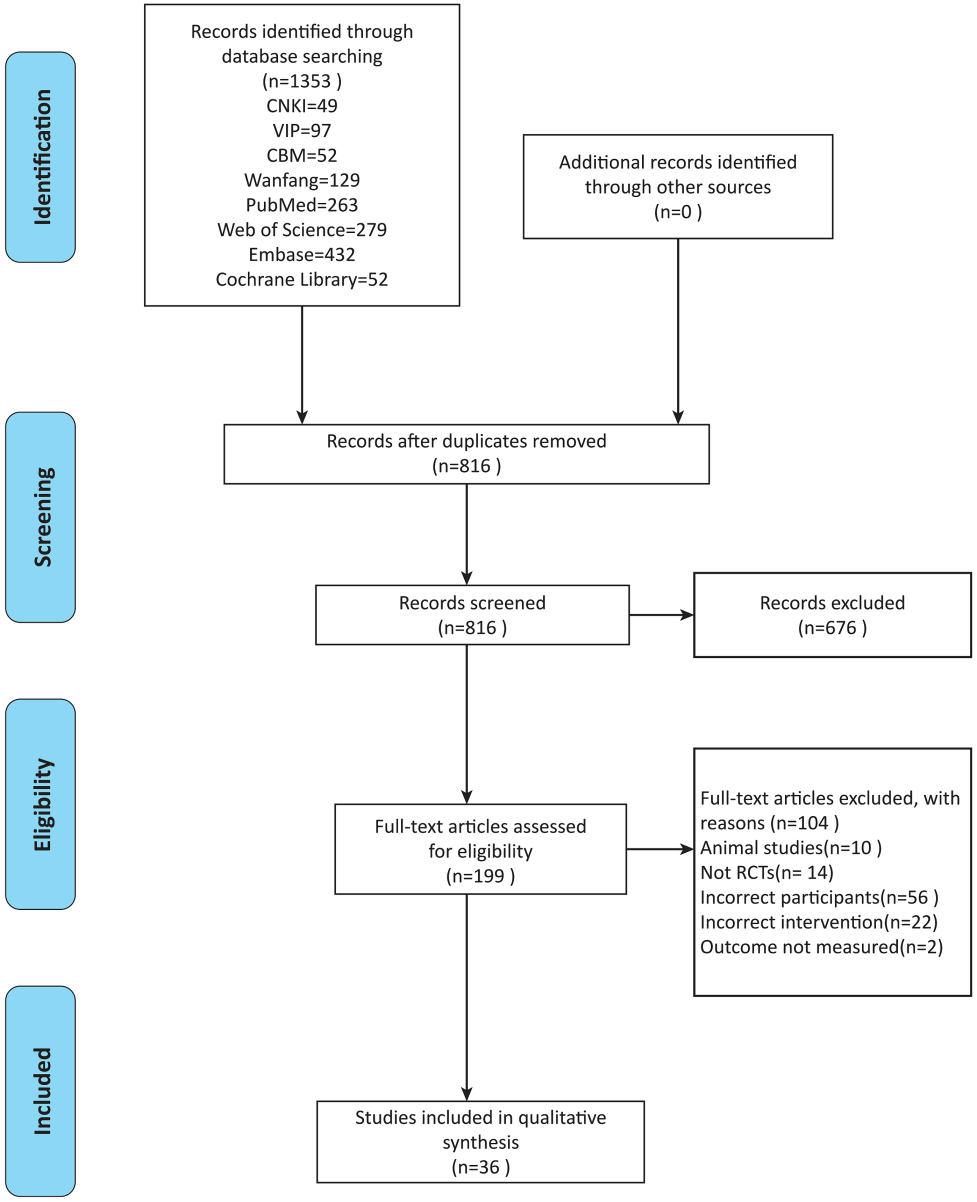
Literature screening flowchart.

A total of 1,353 studies were retrieved from each database, consisting of 49 from CNKI, 129 from Wanfang, 97 from VIP, and 52 from CBM. The English databases yielded 263 studies from PubMed, 432 studies from Embase, 52 studies from Cochrane, and 279 studies from Web of Science. After rigorous screening, a total of 36 studies, comprising 9 in English and 27 in Chinese, were included for the final analysis (16–51) (Figure 1).

### 3.2 Overview of the studies

The literature was synthesized. A total of 2,739 patients with PDN were included, with 1,393 patients treated in the experimental group and 1,346 patients allocated to the control group. The control group received either sham acupuncture or western drugs or usual care, while the trial group received either electroacupuncture or manual acupuncture (Table 1).

**Table 1:** Basic information of included literature.

Included studies	Sample/case	Age/year		Intervention		Diagnosis	Course of treatment	Outcome
	T/C	T	C	T	C			
Adam P 2014	24/21	68.0 ± 11.1	63.0 ± 10.8	Acupuncture	Sham Acupuncture	Clinical Diagnosis of Diabetic Peripheral Neuropathy	70 days	②④⑧
Cao B L 2015	31/29	58.5 ± 9.2	60.83 ± 9.5	Acupuncture	Gliclazide extended release tablets/60-90mg/qd	Needle-like Pain in Extremity; TCM Syndrome Differentiation and Treatment	56 days	①②
					Oral mecobalamine/500μg/tid			
Chen S M 2022	30/30	68.7 ± 9.6	67.2 ± 9.3	Acupuncture; Acupoint-Injection Therapy	Hypoglycemic agents; Insulin; Antihypertensive drugs;	<China Guideline for Type 2 Diabetes (2017)>	14 days	②⑤
					Simvastatin/20mg/qd or Atorvastatin/20mg/qd			
					Aspirin enteric-coated tablets/100mg/qd	Different degrees of skin and limb pain		
					Oral mecobalamine/500μg/tid			
Chi Y R 2017	20/20	NA	NA	Acupuncture	Oral mecobalamine/500μg/tid	NA	30 days	①
					Vitamin B1/1.2mg/tid			
Daichi K 2012	20/18	58.7 ± 10.7	55.9 ± 14.4	Electroacupuncture	NSAID/NA/NA; Vitamin B1/NA/NA	Diabetes Diagnostic Criteria	90 days	①
Deng X M 2021	30/30	59.0 ± 9.0	57.0 ± 9.0	Acupuncture	Pregabalin Capsules/75mg/bid	<China Guideline for Type 2 Diabetes(2017)>	28 days	①③
Fei D D 2010	48/42	60.0 ± 13.8	60.0 ± 13.8	Acupuncture; Moxibustion	Tetramethylpyrazine and Insulin (Intravenous Injection)	Diabetes Mellitus Diagnosis (WHO 1999 criteria)	28 days	②③④⑦
					Mecobalamin Tablets/500μg/qd			
Gong Y 2012	40/40	57.2 ± 8.8	56.3 ± 8.4	Electroacupuncture; Duloxetine Hydrochloride/20-40mg/qd	Oral mecobalamine (Intramuscular Injection)/500μg/qd	Diabetes Mellitus Diagnosis (WHO 1999 criteria)	28 days	①②⑦
He P W 2017	40/40	51.7 ± 9.2	49.2 ± 6.7	Electroacupuncture	Pain Relief Therapy; Nutritional Neurotherapy; Hypoglycemic and Lipid-lowering	Diabetes Mellitus Diagnosis (WHO 1999 criteria)	42 days	②③⑤⑥
						<Guiding Principles for Clinical Research of New Drugs of TCM>		
Hou S K 2018	40/40	54.9 ± 10.1	59.8 ± 4.9	Acupuncture	Hypoglycemic and Lipid-lowering; Vitamin B1/10mg/tid	<China Guideline for Type 2 Diabetes(2013)>	30 days	①②③
					Mecobalamin Tablets/500μg/tid			
Hu J 2014	40/40	55.8 ± 10.1	56.6 ± 9.8	Warmacupuncture	Tetramethylpyrazine and Insulin (Intravenous Injection)	<Diabetes and its complications Chinese and Western medicine diagnosis and treatment>	28 days	①
					Mecobalamin Tablets/500μg/qd			
					Gabapentin/300mg/tid			
Imran H 2014	62/50	51.5 ± 10.5	51.7 ± 11.4	Laser Acupuncture Treatment	Sham Points Treatment	Clinical Diagnosis of Diabetic Peripheral Neuropathy	56 days	②④
Kong X Q 2015	40/40	NA	NA	Warmacupuncture	Tetramethylpyrazine and Insulin (Intravenous Injection)	<Diabetes and its complications Chinese and Western medicine diagnosis and treatment>	28 days	①
					Mecobalamin Tablets/500μg/qd; Gabapentin/300mg/tid			
Kyung S 2018	54/44	NA	NA	Electroacupuncture	Education on diet and lifestyle modification for diabetes	6-month history of PDN and mean weekly pain score of 4 on PINRS	56 days	①⑦
Li F R 2013	50/50	54.1 ± 8.1	51.1 ± 9.4	Acupuncture	Methylcobalamin Injection/1,000μg/qd	Diabetes Mellitus Diagnosis (WHO 1999 criteria)	35 days	①②
						<Diabetes and its complications Chinese and Western medicine diagnosis and treatment>		
Li H X 2018	100/100	55.2 ± 7.8	54.4 ± 8.9	Acupuncture	Vitamin B1/10mg/tid; Methylcobalamin Injection/1,000 μg/qd	Diabetes Mellitus Diagnosis (WHO 1999 criteria)	21 days	①②③
						<Diabetes and its complications Chinese and Western medicine diagnosis and treatment>		
Li N 2019	50/50	57.1 ± 10.2	57.5 ± 10.5	Acupuncture; Bloodletting	Insulin (Intravenous Injection); Exercise and Diet Control	NA	28 days	②
Liu F Q 2016	28/28	46.2 ± 15.3	43.3 ± 13.4	Electroacupuncture	Nutritional Neurotherapy; Hypoglycemic	<Consensus on the diagnosis and treatment of painful peripheral neuropathy>	28 days	②⑦⑧
					Pregabalin/75–150mg/bid	<China Guideline for Type 2 Diabetes (2013)>		
Liu G Q 2022	30/30	59.5 ± 3.0	59.3 ± 2.7	Acupuncture; Moxibustion	Lipoic Acid Injection/250mL/qd; Mecobalamin Tablets/500μg/tid	NA	21 days	①
Liu H Y 2023	30/30	47.0 ± 5.9	45.2 ± 6.4	Acupuncture; Acupressure	Acupressure	<China Guideline for Type 2 Diabetes (2020)>	14 days	①②⑤⑥
						<TCM Clinical Diagnosis and Treatment Guidelines for Diabetic Peripheral Neuropathy(2016)>		
Liu J W 2022	30/30	56.0 ± 5.8	55.7 ± 5.5	Acupuncture	Pregabalin/75mg/bid	<China Guideline for Type 2 Diabetes (2013)>	168 days	①③⑥
						<Guidelines for Prevention and Treatment of Diabetes with TCM>		
Maria T 2019	26/14	60.7 ± 11.8	61.0 ± 9.8	Acupuncture	Usual care	Either an ICD9 250* or ICD10 E11.9, the diagnostic codes for diabetes mellitus, or two emoglobin A1c values > 6.5	84 days	②
Mohamed A 2000	25/25	56.0 ± 8.0	54.0 ± 9.0	Electroacupuncture	Sham Acupuncture	Abnormal Nerve Conduction Study	21 days	②④
Pan Q 2015	36/41	63.2 ± 11.5	65.7 ± 10.2	Electroacupuncture; Intradermal Acupuncture	Insulin and Alprostadil (Intravenous Injection); Exercise and Diet Control	Diabetes Mellitus Diagnosis (WHO 1999 criteria)	21 days	②④
						<Guidelines for Prevention and Treatment of Diabetes with TCM>		
Shevtsova G 2021	66/66	NA	NA	Acupuncture; Gabapentin/900mg/qd	Gabapentin/900mg/qd	NA	60 days	②
Song L 2021	32/32	60.0 ± 18.3	59.0 ± 17.4	Intradermal Acupuncture	Mecobalamin Tablets/500μg/tid	<China Guideline for Type 2 Diabetes(2017)>	56 days	①②
						<TCM diagnosis and treatment programs for 95 diseases in 22 specialties>		
Wang R 2019	50/46	60.3 ± 8.7	61.6 ± 7.3	Acupuncture	Mecobalamin Tablets/500μg/tid	Clinical Diagnosis of Diabetes	60 days	①②③⑤
						Clinical Diagnosis of Diabetic Peripheral Neuropathy		
Yang D L 2017	36/36	54.3 ± 7.3	54.0 ± 7.2	Acupuncture	Epalrestat Tablets/50mg/tid	Clinical Diagnosis of Diabetes	14 days	③
					Mecobalamin Tablets/500μg/tid			
Yang Q 2019	48/48	NA	NA	Acupuncture	Mecobalamin Tablets/500μg/tid	Clinical Diagnosis of Diabetic Peripheral Neuropathy	30 days	①②⑧
					Vitamin B1/1.2mg/tid			
Yang Z 2021	30/30	71.2 ± 4.4	72.2 ± 5.1	Acupuncture	Mecobalamin Tablets/500μg/tid	Clinical Diagnosis of Diabetic Peripheral Neuropathy	14 days	②
					Vitamin B1/1,000mg/tid			
Zagorulko O 2011	20/20	NA	NA	Acupuncture; Conventional Therapy	Conventional Therapy (preparaions of thioctic acid, vitamins with antioxidant and neurotropic action)	NA	20 days	②
Zhang P 2018	41/41	56.3 ± 7.3	56.0 ± 7.2	Acupuncture	Chinese Medicine Therapy	<Clinical Neurology>	60 days	①③
						<Criteria for Diagnosis and Curative Effect of TCM Syndrome>		
Zhang P X 2019	51/51	58.9 ± 6.8	58.9 ± 6.8	Acupuncture	Exercise and Diet Control; Mecobalamin Tablets/500μg/tid	<China Guideline for Type 2 Diabetes (2017)>	28 days	③
						<Guiding Principles for Clinical Research of New Drugs of TCM>		
Zhang Q F 2015	40/40	58.9 ± 6.6	59.7 ± 6.8	Intradermal Acupuncture; Epalrestat/50mg/tid	Epalrestat/50mg/tid	<China Guideline for Type 2 Diabetes (2012)>	60 days	①
Zhan Y 2014	30/30	56.9 ± 4.4	57.5 ± 4.5	Electroacupuncture	Carbamazepine/100mg/tid; Diclofenac Sodium/25/bid	NA	15 days	①③
					Vitamin B complex/10mg/tid			
Zhu Y P 2016	28/28	NA	NA	Acupuncture	Methylcobalamin Injection (intramuscular injection)/500 μg/qd	Diabetes Mellitus Diagnosis (WHO 1999 criteria)	30 days	①

Outcome: ① Total effective rate. ② Pain intensity. ③ MCV, SCV. ④ Depression. ⑤ TCSS. ⑥ TCM syndrome score. ⑦ Adverse events. ⑧ Quality of life.

### 3.3 Risk of bias assessment

**Figure 2:**
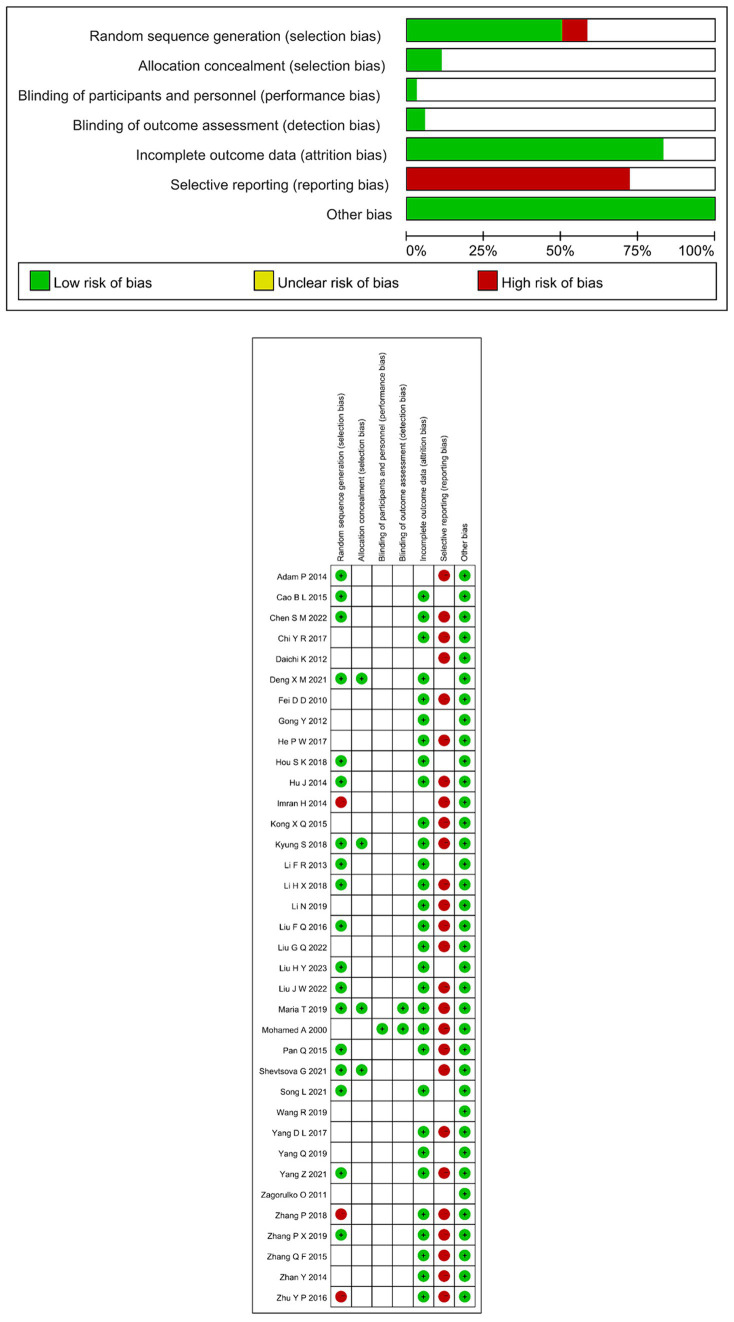
Quality map of included literature.

The outcomes are shown in Figures 2 which shows the percent risk assessment results for each domain. Details of the judgments are presented in Supplementary Table 1. In all studies, there were two domains judged to be at high risk of bias. In 26 studies (16, 18–21, 23–26, 28, 30–34, 37, 39, 41–43, 46–51), the primary problem was due to reporting bias. There are some studies that only report the main findings (i.e., Total effective rate, Pain intensity), secondary findings (i.e., MCV, SCV, Depression, TCSS, Quality of life, TCM syndrome score, Adverse events) were not reported. One study had lower performance bias (47) because they used sham electrical stimulation therapy in the control group, which ensured the participants were blind, while other studies had unclear performance bias. Two studies had low detection bias (47, 49), because the outcome evaluator was not informed about the allocation of intervention, while detection bias in other studies was unclear because there was no indication whether the evaluator was blind. Four studies had low selection bias because random numbers and groupings were kept in sealed opaque envelopes, so the evaluators used a blind approach, while the risk of detection bias was unclear in the other studies. In 18 studies (19, 21, 22, 26, 27, 29, 30, 32, 34, 36–38, 40, 42, 46, 49–51), random table sampling and stratified random sampling were mentioned. Nevertheless, three studies were considered to have high selection bias, since two of the sequences were produced on the basis of admission (23, 33), one is to adopt the method of non-probability sampling (43). The rest of the studies were rated as “unclear risk.” For the assessment of incomplete result data, 30 studies (16–42, 46, 47, 49) were all rated “low risk” and six (43–45, 48, 50, 51) were rated “unclear risk.”

### 3.4 Meta-analysis results

#### 3.4.1 Primary outcomes

##### 3.4.1.1 Total effective rate

**Figure 3:**
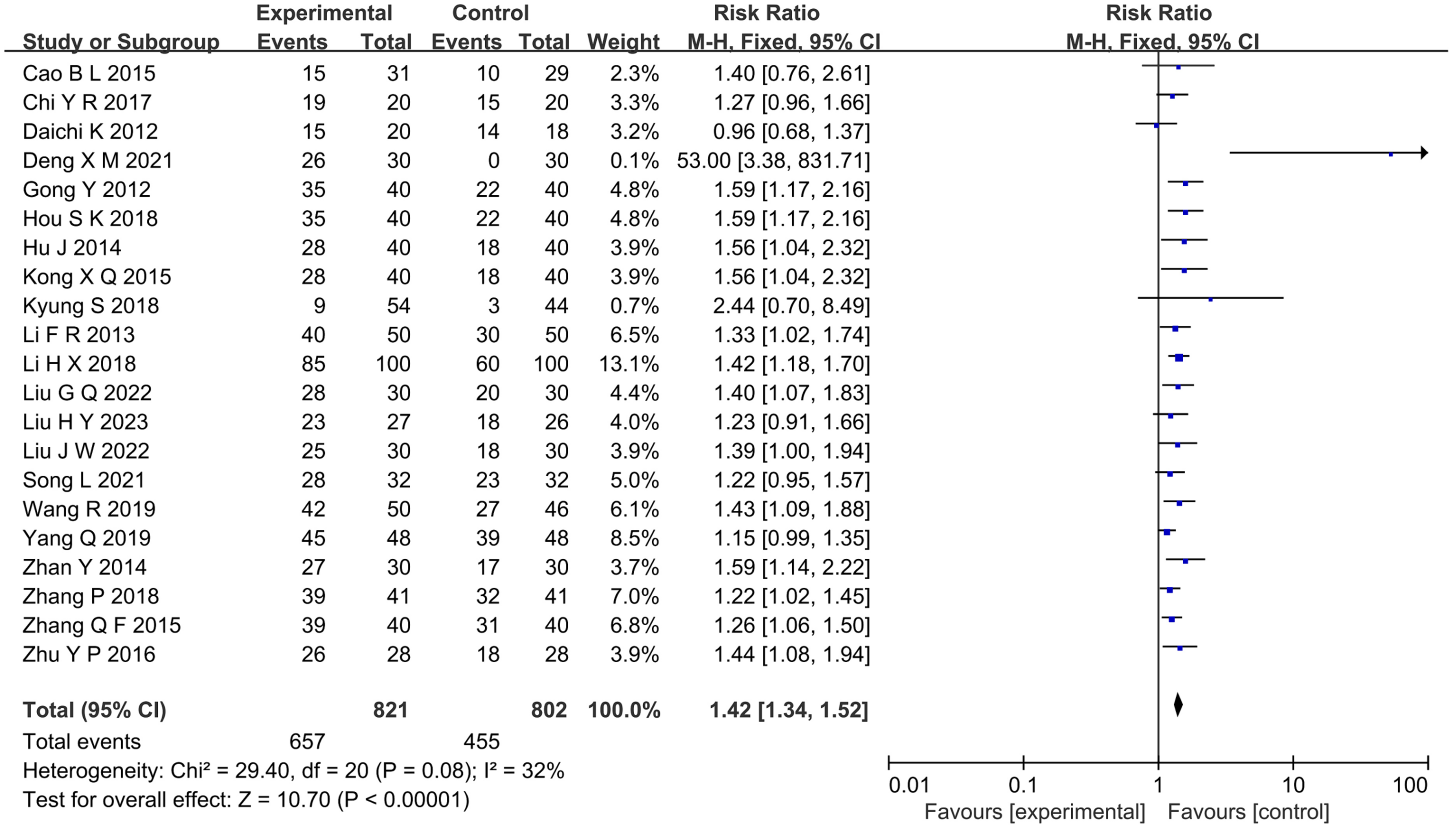
Forest diagram of total effective rate.

Overall response rates were reported in 21 studies comparing the efficacy of acupuncture with MT for diabetic painful neuropathy (16–18, 21–25, 27, 29, 32, 33, 35–38, 40, 41, 44, 46, 48). Outcomes of meta-analysis indicated that the overall efficacy of acupuncture was better than MT (RR = 1.42, 95%CI [1.34, 1.52], *p* < 0.00001; Figure 3).

##### 3.4.1.2 Pain intensity

**Figure 4:**
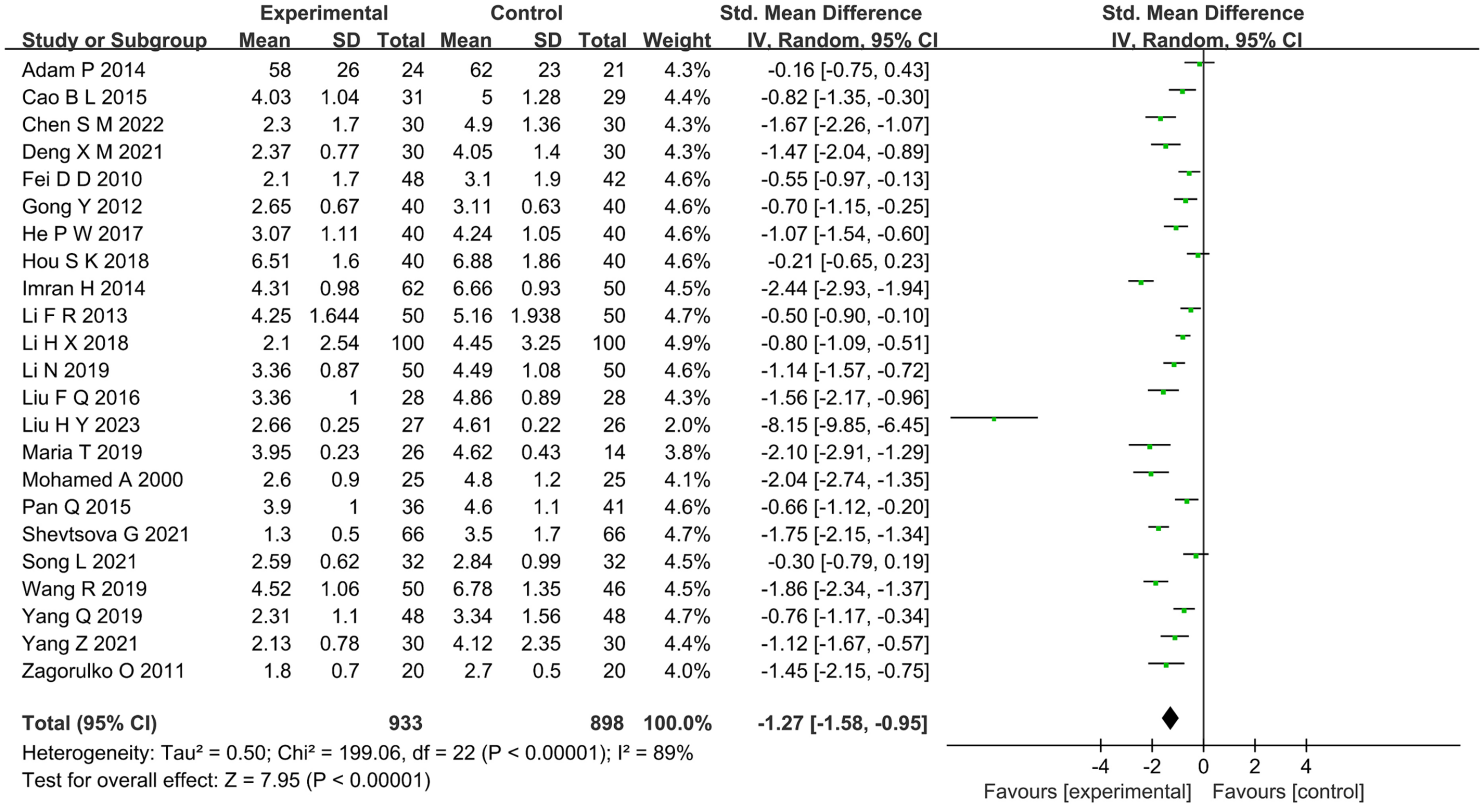
Forest diagram of pain intensity.

The VAS score was used to calculate the pain intensity of acupuncture in patients with diabetes mellitus in 23 studies (17, 19, 22, 27–31, 34–40, 42–45, 47, 49–51). The results of meta-analysis showed that the VAS score of the acupuncture group was statistically different from that of the MT group (SMD =−1.27, 95%CI [−1.58, −0.95], *p* < 0.00001; Figure 4). Compared with the MT group, acupuncture group had better effect on pain relief.

#### 3.4.2 Secondary outcomes

##### 3.4.2.1 MCV, SCV

In 11 studies involving 986 participants, the effects of acupuncture on MCV and SCV caused by sciatica vs. MT were investigated (20, 22, 24, 26, 28, 31–33, 37, 38, 44). The comprehensive results showed that acupuncture improved MCV and SCV significantly better than MT (MD = 3.58, 95%CI [2.77, 4.38], *p* < 0.00001; MD = 3.62, 95%CI [2.75, 4.49], *p* < 0.00001; Supplementary Figure 1).

##### 3.4.2.2 Depression score

There have been five studies on Depression (28, 34, 43, 47, 51). The comprehensive results show that acupuncture is superior to MT in the reduction of diabetic painful neuropathy (SMD = −1.02, 95%CI [1.58, 0.46]) (Supplementary Figure 2).

##### 3.4.2.3 TCSS

Four studies used TCSS score as evaluation index (29–31, 44). Meta-analysis revealed a statistically significant difference in the TCSS score between the acupuncture group and the MT group (MD = −2.41, 95%CI [−3.37, −1.45], *p* < 0.00001; Supplementary Figure 3).

##### 3.4.2.4 Quality of life

Quality of life has 3 studies (17, 19, 51). Combined results show that acupuncture outperforms the MT group in reducing and enhancing the quality of diabetic painful neuropathy (SMD = 1.06, 95%CI [0.66, 1.46]) (Supplementary Figure 4).

##### 3.4.2.5 TCM syndrome score

There are 3 studies on TCM syndrome score (29, 31, 32). The comprehensive results showed that TCM syndrome score in the acupuncture group was statistically different from that in the MT group (MD = −4.99, 95%CI [−6.79, −3.18], *p* < 0.00001; Supplementary Figure 5).

##### 3.4.2.6 Adverse events

There are four studies on Adverse events (19, 28, 35, 46). Combined results showed that the difference of acupuncture was indistinctive in reducing the incidence of adverse reactions in diabetic painful neuropathy compared with MT (RR = 0.88, 95%CI [0.09, 8.24]) (Supplementary Figure 6).

### 3.5 Results of subgroup analysis

#### 3.5.1 Primary outcomes

##### 3.5.1.1 Total effective rate

Subgroup analysis indicated that EA (RR = 1.40, 95%CI [1.03, 1.90]) and MA (RR = 1.42, 95%CI [1.32, 1.52]) were better than MT in improving overall response. Regarding the course of treatment, we found that acupuncture < 30 times (RR = 1.61, 95%CI [1.44, 1.80]) and ≥30 times (RR = 1.31, 95%CI [1.21, 1.42]) were statistically significant improvements in the total response rate compared to MT (Table 2).

**Table 2:** The subgroup analysis for the outcomes of included studies.

Subgroup	Eligible studies	Intervention Group (n)	Control Group (n)	RR/SMD (95%Cl)	*p* value	Heterogeneity test	Effect model
Total effective rate							
Acupuncture categories							
EA vs. MT	4	144	132	1.40[1.03,1.90]	<0.001	*p* = 0.08, I^2^ = 55%	Random
MA vs. MT	17	677	670	1.42[1.32,1.52]	<0.001	*p* = 0.14, I^2^ = 28%	Fixed
Total sessions of treatment							
<30	8	337	336	1.61[1.44,1.80]	<0.001	*p* = 0.09, I^2^ = 43%	Fixed
Greater than or equal to 30	13	484	466	1.31[1.21,1.42]	<0.001	*p* = 0.59, I^2^ = 0%	Fixed
Pain intensity							
Acupuncture categories							
EA vs. MT	6	217	216	−1.05[−1.45, −0.64]	<0.001	*p* = 0.001, I^2^ = 75%	Random
MA vs. MT	17	716	682	−1.36[−1.76, −0.96]	<0.001	*p* < 0.001, I^2^ = 91%	Random
Total sessions of treatment							
<30	12	464	462	−1.46[−1.91, −1.01]	<0.001	*p* < 0.001, I^2^ = 89%	Random
Greater than or equal to 30	11	469	436	−1.07[−1.54, 0.61]	<0.001	*p* < 0.001, I^2^ = 90%	Random
MCV							
Acupuncture categories							
EA vs. MT	3	120	114	3.32[2.32, 4.31]	<0.001	*p* = 0.43, I^2^ = 0%	Fixed
MA vs. MT	8	378	374	3.72[2.65, 4.80]	<0.001	*p* = 0.0006, I^2^ = 72%	Random
SCV							
Acupuncture categories							
EA vs. MT	3	120	114	2.79[1.80, 3.77]	<0.001	*p* = 0.25, I^2^ = 27%	Fixed
MA vs. MT	8	378	374	3.93[2.84, 5.03]	<0.001	*p*<0.001, I^2^ = 83%	Random
Depression							
Acupuncture categories							
EA vs. MT	4	171	158	−5.16[−8.57, −1.75]	0.003	*p*<0.001, I^2^ = 93%	Random
MA vs. MT	1	24	21	−12.60[−16.56, −8.64]	<0.001	NA	Random

RR, risk ratio; CI, confidence interval; MT, medicine treatment; MA, manual acupuncture; EA, electroacupuncture; NA, not applicable.

##### 3.5.1.2 Pain intensity

The subgroup analysis showed that the VAS score was lower than MT (SMD = −1.05, 95%CI [−1.45, −0.64]) and MA (SMD = −1.36, 95% confidence interval [−1.76, −0.96]). However, there was a high degree of heterogeneity (I^2^ = 91%) when comparing MA vs. MT. Regarding the duration of acupuncture treatment, we found <30 days (SMD = −1.46, 95%CI [−1.91, −1.01]) and 30 days or more (SMD = −1.07, 95%CI [−1.54, 0.61]) was more effective on reducing VAS score than MT (Table 2).

#### 3.5.2 Secondary outcomes

##### 3.5.2.1 MCV, SCV

Subgroup analysis showed that EA (MD = 3.32, 95%CI [2.32, 4.31]), MA (MD = 3.72, 95%CI [2.65, 4.80]) and EA (MD = 2.79, 95%CI [1.80, 3.77]), MA (MD = 3.93, 95%CI [2.84, 5.03]) were better than MT in improving MCV and SCV, respectively (Table 2).

##### 3.5.2.2 Depression score

Subgroup analysis showed that EA (MD = −5.16, 95%CI [−8.57, −1.75]) and MA (MD = −12.60, 95%CI [−16.56, −8.64]) were superior to MT in reducing anxiety and depression in patients (Table 2).

### 3.6 Sensitivity analysis

Heterogeneity was tested for eight major outcomes using RevMan (version5.4). So as to verify the stability of the outcomes, a sensitivity analysis was also performed to eliminate the included references one by one and to analyze other studies to estimate whether the results would be significantly affected by a single study. In the heterogeneity test of the total effective rate, *p* > 0.05, I^2^ < 50%, which can be considered to be within a reasonable range of heterogeneity. What’s more, in sensitivity analysis, there was no significant change in the overall efficacy effect of the exclusion of studies alone, but heterogeneity decreased to 0 when excluding one study (38). In the heterogeneity test of MCV and SCV, I^2^ = 63% of MCV data and I^2^ = 63% of SCV data both show high heterogeneity. Sensitivity analysis was performed, and when a study was excluded, heterogeneity returned to the reasonable range, with I^2^ < 50% (32). TCM (I^2^ = 82%) was highly heterogeneous. Sensitivity analysis was performed. When a study was excluded, I^2^ was reduced to 0 and no heterogeneity was observed. The preliminary conclusion is that the study is associated with heterogeneity (29). In the heterogeneity test of pain degree score, *p* < 0.001, I^2^ = 89%, showing great heterogeneity. The sensitivity analysis was performed by single elimination method, and the effect value did not change significantly, nor did the heterogeneity change significantly. The results showed good stability. Depression score (I^2^ = 84%), TCSS (I^2^ = 76%), QOL (I^2^ = 99%), Adverse events (I^2^ = 76%) are highly heterogeneous. The sensitivity analysis was carried out by one-by-one exclusion method, and the effect value and heterogeneity did not change significantly (Supplementary Table 2).

### 3.7 Publication bias

Funnel plots were generated to assess the publication bias to various indicators, namely Total effective rate, Pain intensity, MCV, SCV, Depression score, TCSS, Quality of life, TCM syndrome score and Adverse events (Supplementary Figure 7). The results showed that there was evidence of publication bias in total efficacy, pain intensity, MCV, and SCV (*p* < 0.05). This observation could be attributed to the limited sample size of the incorporated research. Despite the presence of publication bias, the effect sizes were adjusted, and the outcomes continued to be statistically significant before and after adjustment for each outcome indicator. This suggests that publication bias minimally affected the outcomes, and consequently, the stability of the findings was maintained. The results of Egger’s test are presented in Supplementary Table 3 and Supplementary Figure 8, with Supplementary Table 4 and Supplementary Figure 9 showing the Trim and Filling method.

### 3.8 TSA analysis results

In terms of Total effective rate, the TSA result shows that the Z-curve has crossed both the traditional level of statistical significance and the TSA threshold (Supplementary Figure 10), indicating that although the cumulative amount of information has not reached the anticipated figure, no additional experiments are required to reach a favorable outcome preemptively. As for depression scores, the curve does not cross the TSA threshold. This implies insufficient evidence supporting a notable impact on depression scores. And the other outcomes (Pain Intensity, MCV, SCV) all passed the traditional and TSA thresholds, pointing to an adequate number of studies to underpin our conclusions.

## 4 Discussion

Our synthesis of evidence from 36 studies involving 2,739 PDN patients underscored the potential of acupuncture as an alternative to conventional pharmacological treatments. This study used evidence-based medicine to systematically review and Meta-analyze clinical studies related to the combination of Chinese and Western medicine using acupuncture for the treatment of PDN in order to evaluate acupuncture’s efficacy and safety as a supplementary therapy for PDN.

Meta-analysis results showed that the total effective rate of acupuncture therapy for PDN was significantly higher than that of MT treatment, which was also characterized by a significant reduction in Pain intensity, and was significantly better in improving MCV and, SCV, diabetic painful neuropathies, and quality of life, and scored better in TCSS and TCM scores. In this criterion, acupuncture and MT had been found to be more effective in reducing the adverse events of painful diabetic neuropathies. Statistically, there was no significant difference in adverse effects of diabetic painful neuropathy in comparison with acupuncture and MT. From another perspective, the findings demonstrated that acupuncture is a promising PDN treatment with notable efficacy and a good safety profile. In addition, in the heterogeneity test of pain degree score, *p* < 0.001, I^2^ = 89%, showing a large heterogeneity. The sensitivity analysis was carried out by one-by-one exclusion method. The effect size did not change significantly, and the heterogeneity did not change significantly, and the results showed good stability. Consequently, we hypothesized that sample heterogeneity, variations in measurement instruments, and variations in study design may be the cause of the high heterogeneity and that it was not caused by literature bias factors. The study’s findings suggested that acupuncture was a promising treatment option for PDN, with significant efficacy and a favorable safety profile.

At present, the pathogenesis and pathogenesis of PDN were not well defined. It was now generally accepted that the pathophysiology of PDN may be related to changes in blood glucose and changes in sodium channels (52). Other potential mechanisms included increased glycemic instability in the development of neuropathic pain (53), increased peripheral nerve peripheral blood flow (54), altered microcirculation in the skin of the foot (55), decreased intraepidermal nerve fiber density in early neuropathy (56), increased thalamic vascularity (57), and autonomic dysfunction (58). Pain intensity in PDN was moderate to severe with exacerbations typically experienced during the night, affecting quality of sleep (59). The paroxysmal effects of acupuncture could be very helpful in relieving this discomfort. Local nerves were impacted by acupuncture, which decreased the nerves’ sensitivity to direct pain. Peripheral insertion of an acupuncture needle into a specific point caused “Deqi” sensation and afferent fibers stimulation (60, 61). This nerve stimulation was conducted via interneurons and sensory ganglia to the spinal cord, which could modulate brainstem motor neurons activity, activate the opioid receptors and induce following analgesic effects (62). A comprehensive analysis of various clinical trials on treatment approaches for PDN declared acupuncture as a supplementary treatment with few adverse effects for PDN patients, supported by moderate evidence (2B+ or 1B+), primarily because of methodological and blinding issues (63).

According to the review by Eunwoo Cho and Woojin Kim (63), the mechanism for acupuncture action was channeled through multiple pathways and may, therefore, relate to a variety of molecules in the peripheral nerves (e.g., P65, CBS, P2X3R p-PKC GPR78, and caspase-12) and in the spinal cord (e.g., NGF, SP, TRPV1, P2X4, OX42, and GAD67) (63). Studies had shown that ST36 appears to be more effective in reducing pain and increasing nerve conduction velocity, and has been shown to be effective in treating a variety of neuropathic pain (64, 65). ST36, BL23 and SP6 are also widely used in the treatment of diabetes (66). EA acting on SP9 has significant thermal and mechanical analgesic effects (67). The commonly used acupoints included Foot Sanli (ST36), Great Spine (BL13), Large Intestine Yu (BL20), Sanyinjiao (SP6), and Sea of Blood (SP9). These results were considered sufficient to prove the analgesic efficacy of acupuncture and to indicate that acupuncture treatment may be an effective and specific with respect to control intervention complementary and alternative method for the treatment of PDN.

Due to long-term chronic pain, more than two thirds of patients with PDN suffered from mental symptoms such as anxiety and depression of different degrees (4), which brought great pain to the patients. The decrease in scores for depression associated with PDN was another significant secondary finding since depression is highly prevalent in the populations suffering from chronic pain. Impact on TCSS and quality of life further supported the multifaceted benefits on these patients afforded by acupuncture.

Furthermore, acupuncture was a safe alternative to or addition to traditional pharmaceutical treatments due to its similar safety profile to that of MT, which showed no discernible difference in the rate of adverse events. This was the same with the findings of Dania Blaibe et al. (68). In the words of Dania Blaibe, acupuncture was a non-invasive and cost-effective therapy with reasonable safety profile. In a meta-analysis of data involving 20,827 patients from 39 trials, it was shown that acupuncture was superior to both the sham and no acupuncture controls for each pain condition (69).

Within the analysis of subgroups, every acupuncture technique and session duration method surpassed MT, indicating the potential superiority of acupuncture as a therapeutic modality. However, the analysis also revealed a high degree of heterogeneity between MA and MT in terms of pain intensity. Such heterogeneity may be due to the greater variability in application techniques, depth of needle, and duration of acupuncture in MA compared to the more precisely controlled amount of needle stimulation in EA. The observed heterogeneity between manual acupuncture (MA) and electroacupuncture (EA) may stem from the lack of standardized protocols across studies, particularly in terms of application techniques, needle depth, and duration of treatment. This variability highlighted a critical challenge in developing acupuncture as a standardized medical practice. Without such standards, it may be difficult to compare and generalize findings across different studies and practitioners. Thus, establishing uniform criteria for evaluating acupuncture treatments and outcomes is essential to ensure the reliability, validity, and reproducibility of research findings.

Moreover, the study revealed that only a small fraction of the analyzed studies demonstrated low biases in selection, performance, and detection. This highlights the pressing need for more rigorous research methodologies in acupuncture studies. Blinding techniques and standardized controls remain technically challenging in acupuncture practice, while their implementation is of primordial importance for validity and reliability of study results. Researchers should prioritize overcoming these challenges and incorporate blinding methods into future RCT designs to enhance the credibility and quality of their investigations.

Due to the inclusion of Chinese databases in this study, there is a potential limitation due to the different quality of manuscripts in Chinese databases journals. To mitigate these risks, future studies should focus on incorporating internationally recognized databases and ensure rigorous quality control when selecting sources. Importantly, the bulk of the examined studies paid little attention to secondary outcomes, which raises questions regarding the comprehensive assessment of acupuncture’s effects on PDN. To validate and strengthen the conclusions drawn in this study, it is imperative to conduct multi-center trials with large sample sizes and minimal biases. Such studies will help establish a more reliable and relevant evidence base, guiding clinicians and policymakers in making informed decisions regarding the integration of acupuncture into the comprehensive management of PDN.

## 5 Conclusion

In conclusion, the study’s findings suggest that acupuncture is a promising treatment option for PDN, with significant efficacy and a favorable safety profile. Acupuncture demonstrates significant effectiveness in improving PDN outcomes, including Total effective rate, Pain intensity, MCV, SCV, Depression score, TCSS, Quality of life, TCM syndrome score. But the Adverse events rate is no different in trail group and control group.

## Data availability statement

The original contributions presented in the study are included in the article/Supplementary material, further inquiries can be directed to the corresponding author.

## Author contributions

JL: Conceptualization, Data curation, Formal analysis, Methodology, Resources, Software, Writing – original draft. YL: Conceptualization, Data curation, Formal analysis, Writing – original draft. YH: Formal analysis, Investigation, Methodology, Writing – original draft. QY: Data curation, Formal analysis, Validation, Visualization, Writing – original draft. XL: Data curation, Formal analysis, Writing – original draft. YY: Data curation, Formal analysis, Writing – original draft. BZ: Data curation, Formal analysis, Writing – original draft. WS: Conceptualization, Data curation, Formal analysis, Writing – original draft, Writing – review & editing.

## Footnotes

^1^ https://ctu.dk/tsa/
