# Supplementary material for: Efficacy and safety of acupuncture for painful diabetic neuropathy: a systematic review and meta-analysis
Source: Front Neurol. 2024 Jun 5;15:1402458. doi: 10.3389/fneur.2024.1402458 (PMC11188462; doi:10.3389/fneur.2024.1402458)
Supplement: Supplementary file 1 [file Data_Sheet_1.PDF]

Study or Subgroup	Experimental Mean	Experimental SD	Experimental Total	Control Mean	Control SD	Control Total	Weight	Mean Difference IV, Random, 95% CI
**3.1.1 MCV**								
Deng X M 2021	46	2.2	30	43.6	1.6	30	12.8%	2.40 [1.43, 3.37]
Fei D D 2010	45.5	5.4	48	43.1	2.7	42	9.1%	2.40 [0.67, 4.13]
He P W 2017	42.07	3.45	40	38.46	3.71	40	9.9%	3.61 [2.04, 5.18]
Hou S K 2018	46.8	5.14	40	44.5	4.52	40	7.5%	2.30 [0.18, 4.42]
Li H X 2018	58.97	4	100	56.45	4	100	12.1%	2.52 [1.41, 3.63]
Liu J W 2022	56.47	3.46	30	50.14	3.62	30	8.9%	6.33 [4.54, 8.12]
Wang R 2019	52.53	7.82	50	46.21	6.52	46	5.3%	6.32 [3.45, 9.19]
Yang D L 2017	46.43	7.15	36	42.72	7.07	36	4.4%	3.71 [0.43, 6.99]
Zhan Y 2014	47.4	3.1	32	43.4	4.6	32	8.3%	4.00 [2.08, 5.92]
Zhang P 2018	58.11	2.48	41	55.17	2.85	41	11.9%	2.94 [1.78, 4.10]
Zhang P X 2019	59.98	4.12	51	55.13	3.96	51	9.9%	4.85 [3.28, 6.42]
**Subtotal (95% CI)**			**498**			**488**	**100.0%**	**3.58 [2.77, 4.38]**
Heterogeneity: Tau² = 1.08; Chi² = 27.15, df = 10 (P = 0.002); I² = 63%								
Test for overall effect: Z = 8.69 (P < 0.00001)								
**3.1.2 SCV**								
Deng X M 2021	42.9	1.8	30	39.9	1.6	30	11.5%	3.00 [2.14, 3.86]
Fei D D 2010	41.3	3.5	48	39.4	3.7	42	9.4%	1.90 [0.41, 3.39]
He P W 2017	46.72	4.39	40	43.73	4.75	40	7.7%	2.99 [0.99, 4.99]
Hou S K 2018	44.9	5.39	40	41.5	2.27	40	8.3%	3.40 [1.59, 5.21]
Li H X 2018	59.3	4.9	100	56.3	4	100	10.3%	3.00 [1.76, 4.24]
Liu J W 2022	58.89	4.9	30	50.11	3.31	30	7.3%	8.78 [6.66, 10.90]
Wang R 2019	52.34	9.54	50	44.38	8.69	46	4.0%	7.96 [4.31, 11.61]
Yang D L 2017	48.44	2.87	36	45.33	2.96	36	9.9%	3.11 [1.76, 4.46]
Zhan Y 2014	46.2	2.9	32	42.4	4	32	8.6%	3.80 [2.09, 5.51]
Zhang P 2018	44.93	1.86	41	42.75	1.58	41	11.8%	2.18 [1.43, 2.93]
Zhang P X 2019	49.22	2.78	51	45.45	2.02	51	11.2%	3.77 [2.83, 4.71]
**Subtotal (95% CI)**			**498**			**488**	**100.0%**	**3.62 [2.75, 4.49]**
Heterogeneity: Tau² = 1.53; Chi² = 45.53, df = 10 (P < 0.00001); I² = 78%								
Test for overall effect: Z = 8.14 (P < 0.00001)								

Test for subgroup differences: Chi² = 0.01, df = 1 (P = 0.94), I² = 0%

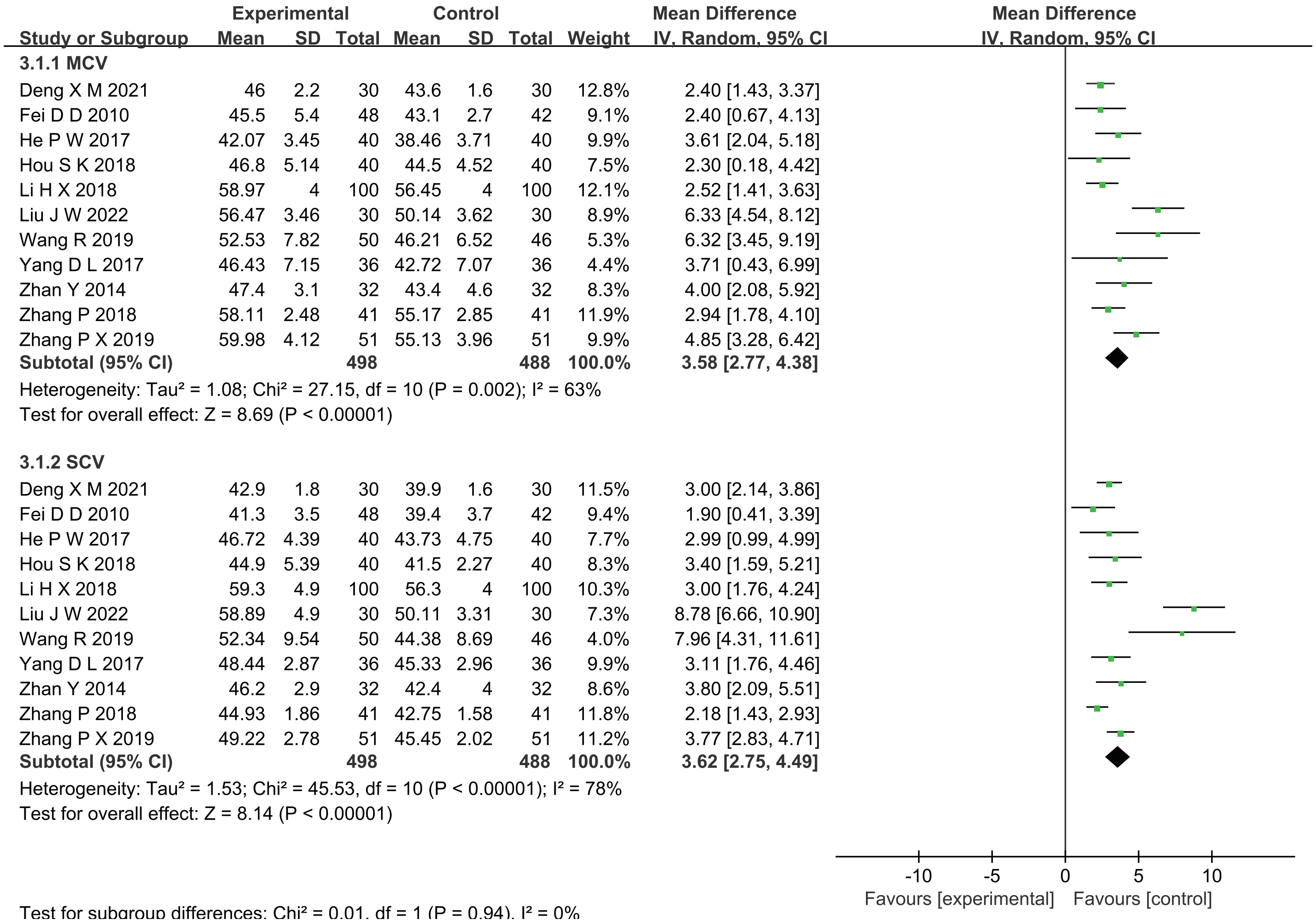

Supplementary Figure 1. Forest plot of MCV, SCV.

Study or Subgroup	Experimental Mean	Experimental SD	Experimental Total	Control Mean	Control SD	Control Total	Weight	Std. Mean Difference IV, Random, 95% CI
Adam P 2014	8.1	4.6	24	20.7	8.2	21	17.5%	-1.90 [-2.61, -1.18]
Fei D D 2010	43.2	11.7	48	52.3	11.2	42	21.4%	-0.79 [-1.22, -0.36]
Imran H 2014	7.41	1.92	62	8.6	2.5	50	22.0%	-0.54 [-0.92, -0.16]
Mohamed A 2000	8.1	4.6	25	20.7	8.2	25	18.1%	-1.87 [-2.54, -1.19]
Pan Q 2015	10	1.6	36	10.9	3.5	41	21.1%	-0.32 [-0.77, 0.13]
**Total (95% CI)**			**195**			**179**	**100.0%**	**-1.02 [-1.58, -0.46]**
Heterogeneity: Tau² = 0.33; Chi² = 24.85, df = 4 (P < 0.0001); I² = 84%								
Test for overall effect: Z = 3.58 (P = 0.0003)								

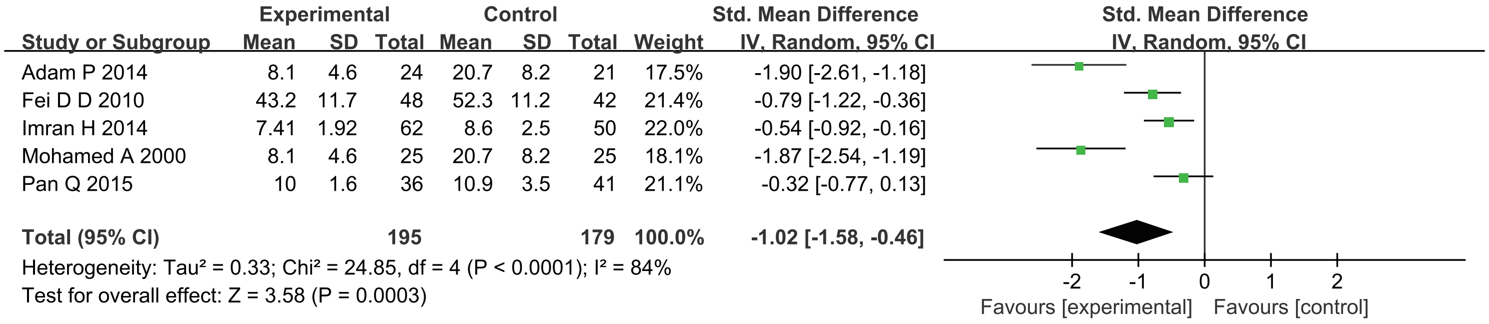

Supplementary Figure 2. Forest plot of Depression score.

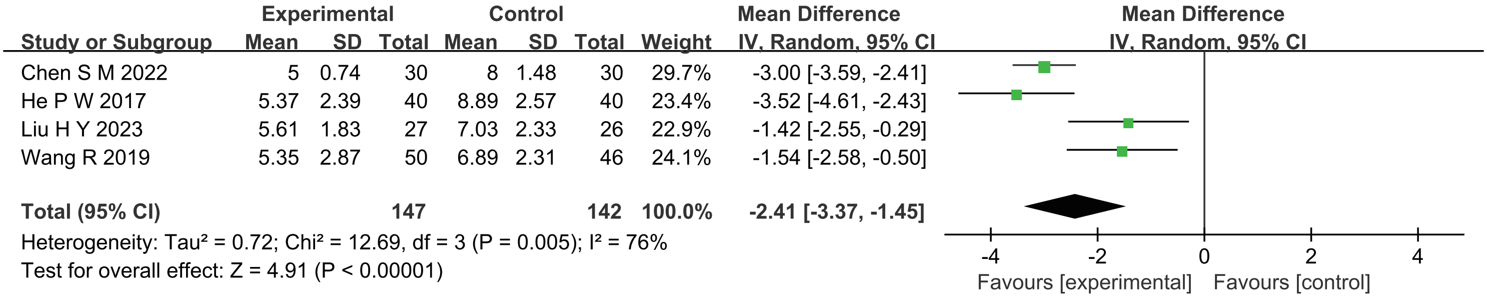

Supplementary Figure 3. Forest plot of TCSS.

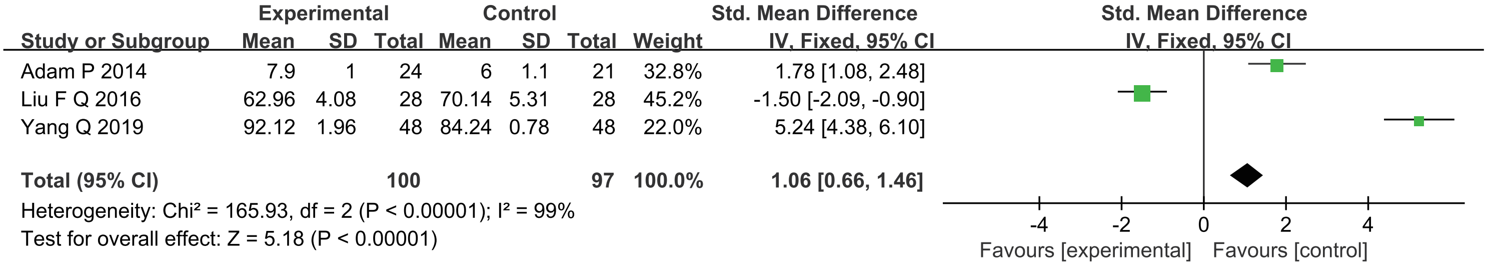

Supplementary Figure 4. Forest plot of Quality of life.

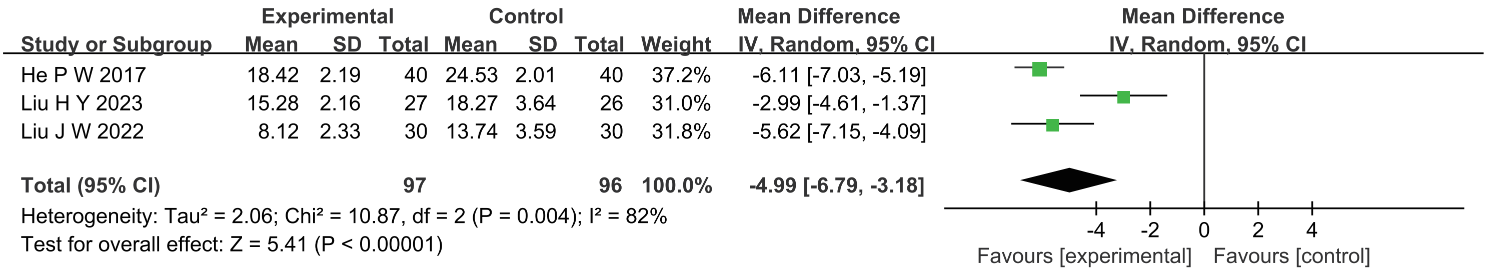

Supplementary Figure 5. Forest plot of TCM syndrome score.

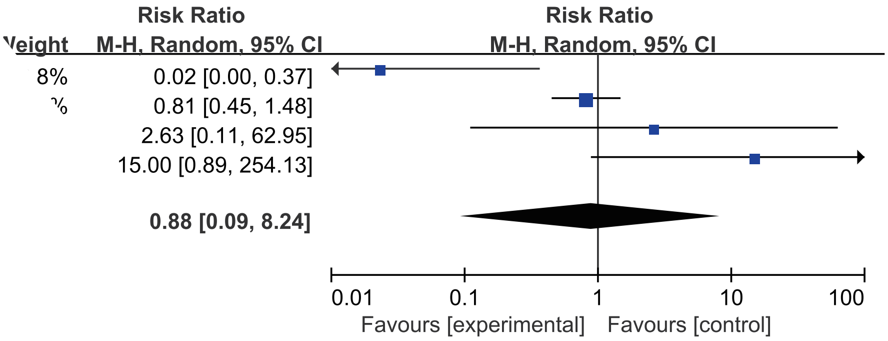

Supplementary Figure 6. Forest plot of Adverse events.

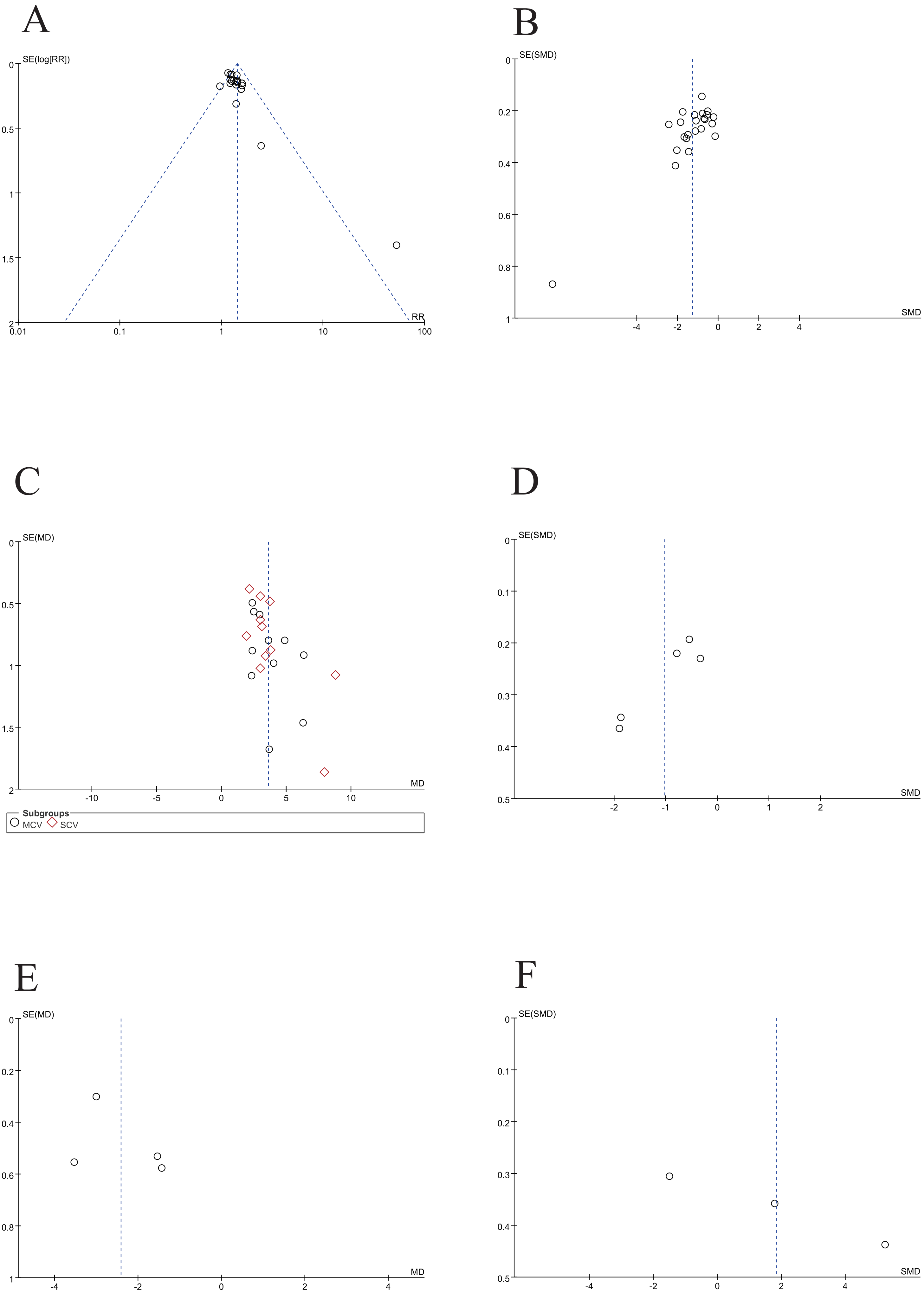
A
SE(log[RR])
RR
0.01
0.1
1
10
100
0
0.5
1
1.5
2
B
SE(SMD)
SMD
-4
-2
0
2
4
0
0.2
0.4
0.6
0.8
1
C
SE(MD)
MD
-10
-5
0
5
10
Subgroups
MCV
SCV
D
SE(SMD)
SMD
-2
-1
0
1
2
0
0.1
0.2
0.3
0.4
0.5
E
SE(MD)
MD
-4
-2
0
2
4
F
SE(SMD)
SMD

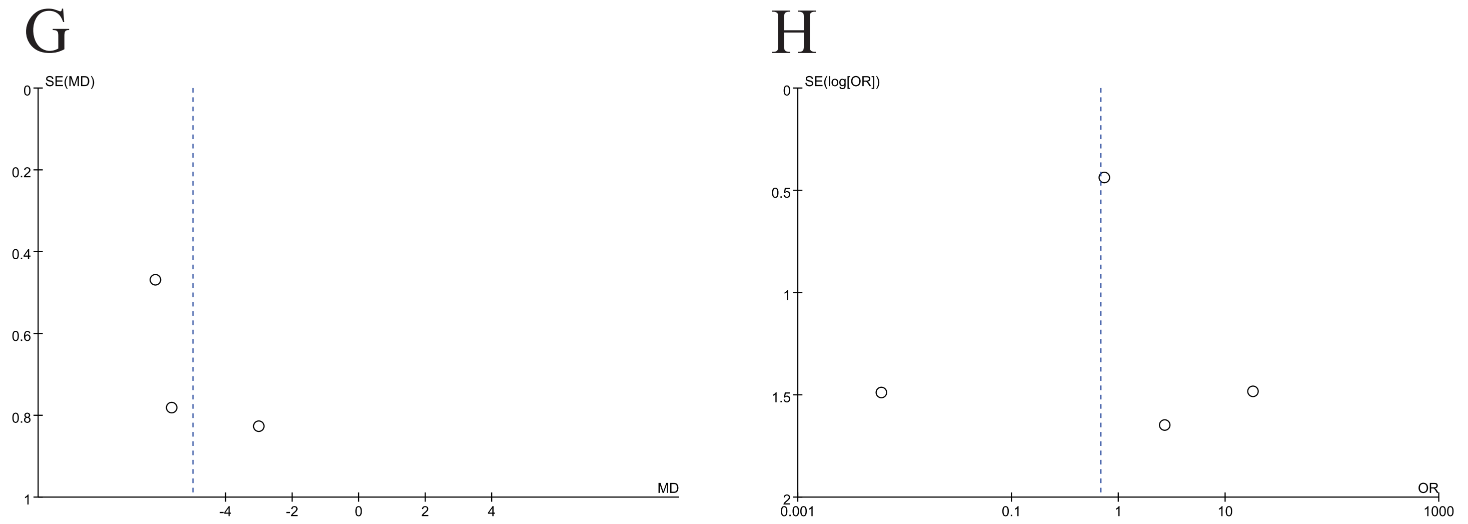

Supplementary Figure 8. Funnel plots. (A) Funnel plot of Total effective rate. (B) Funnel plot of Pain intensity. (C) Funnel plot of MCV, SCV. (D) Funnel plot of Depression score. (E) Funnel plot of TCSS. (F) Funnel plot of Quality of life. (G) Funnel plot of TCM syndrome score. (H) Funnel plot of Adverse events.

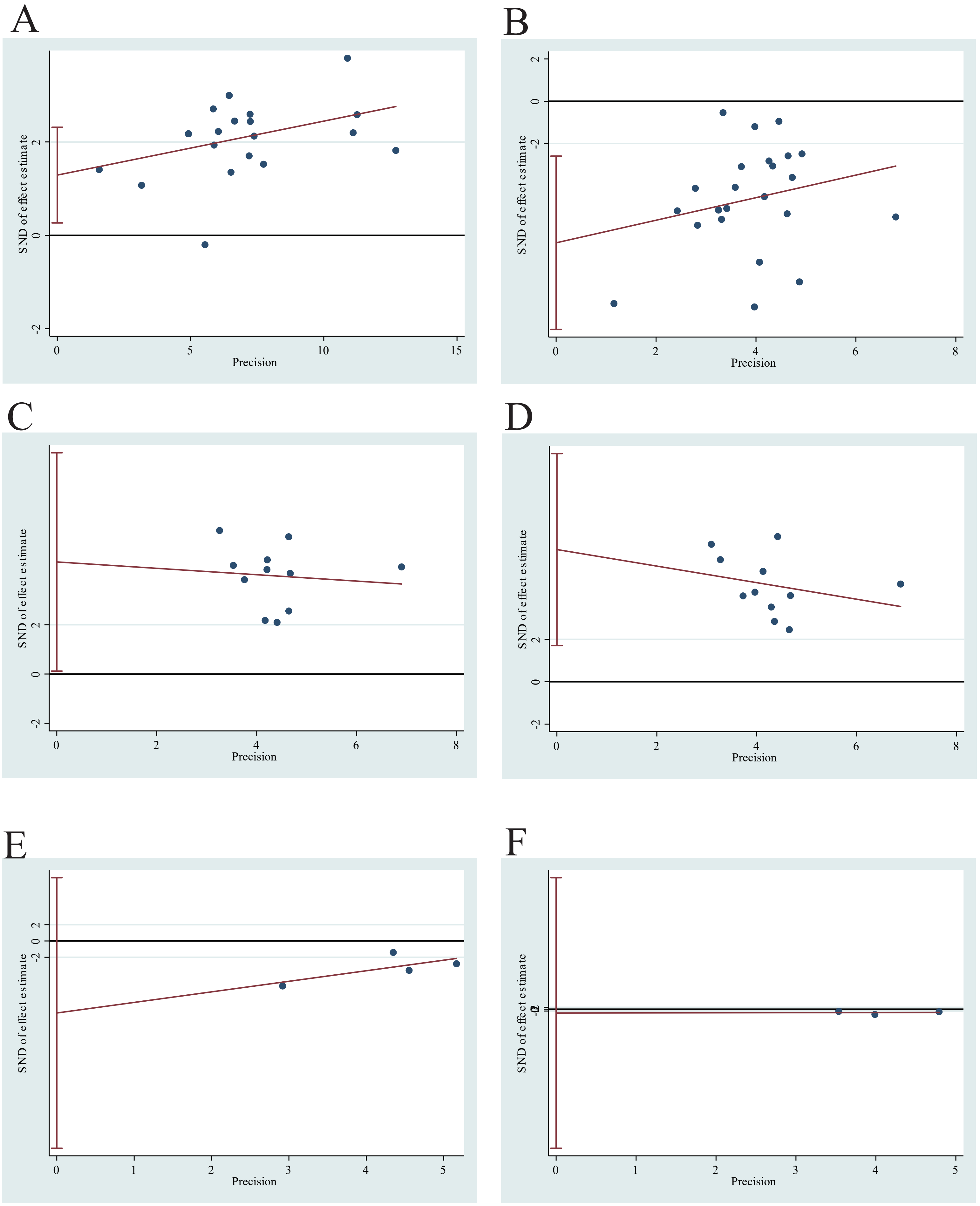
A
B
C
D
E
F
SND of effect estimate
Precision

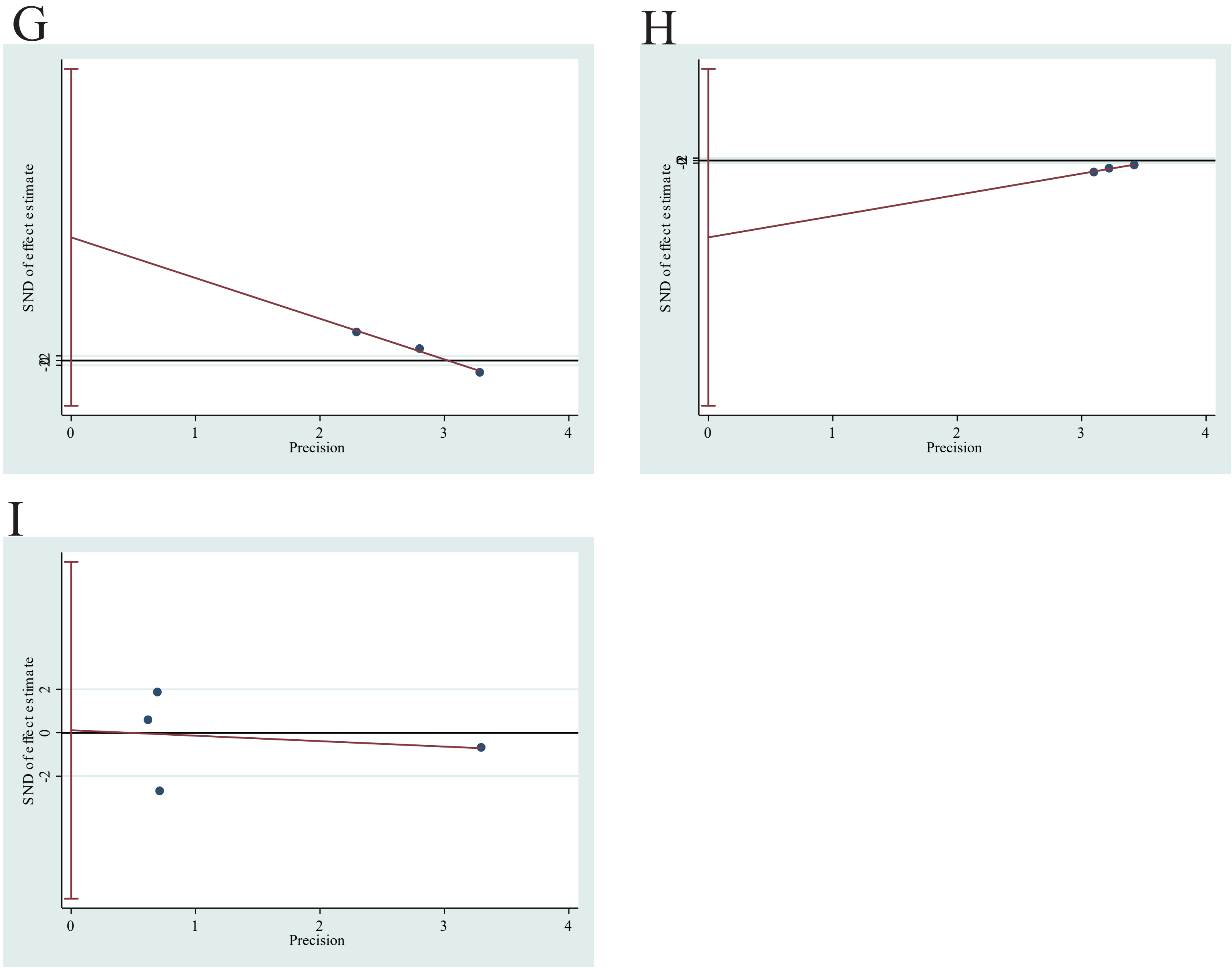

Supplementary Figure 8. Egger's test to assess publication bias. (A) Egger's test of Total effective rate. (B) Egger's test of Pain intensity. (C) Egger's test of MCV. (D) Egger's test of SCV. (E) Egger's test of Depression score. (F) Egger's test of TCSS. (G) Egger's test of Quality of life. (H) Egger's test of TCM syndrome score. (I) Egger's test of Adverse events.

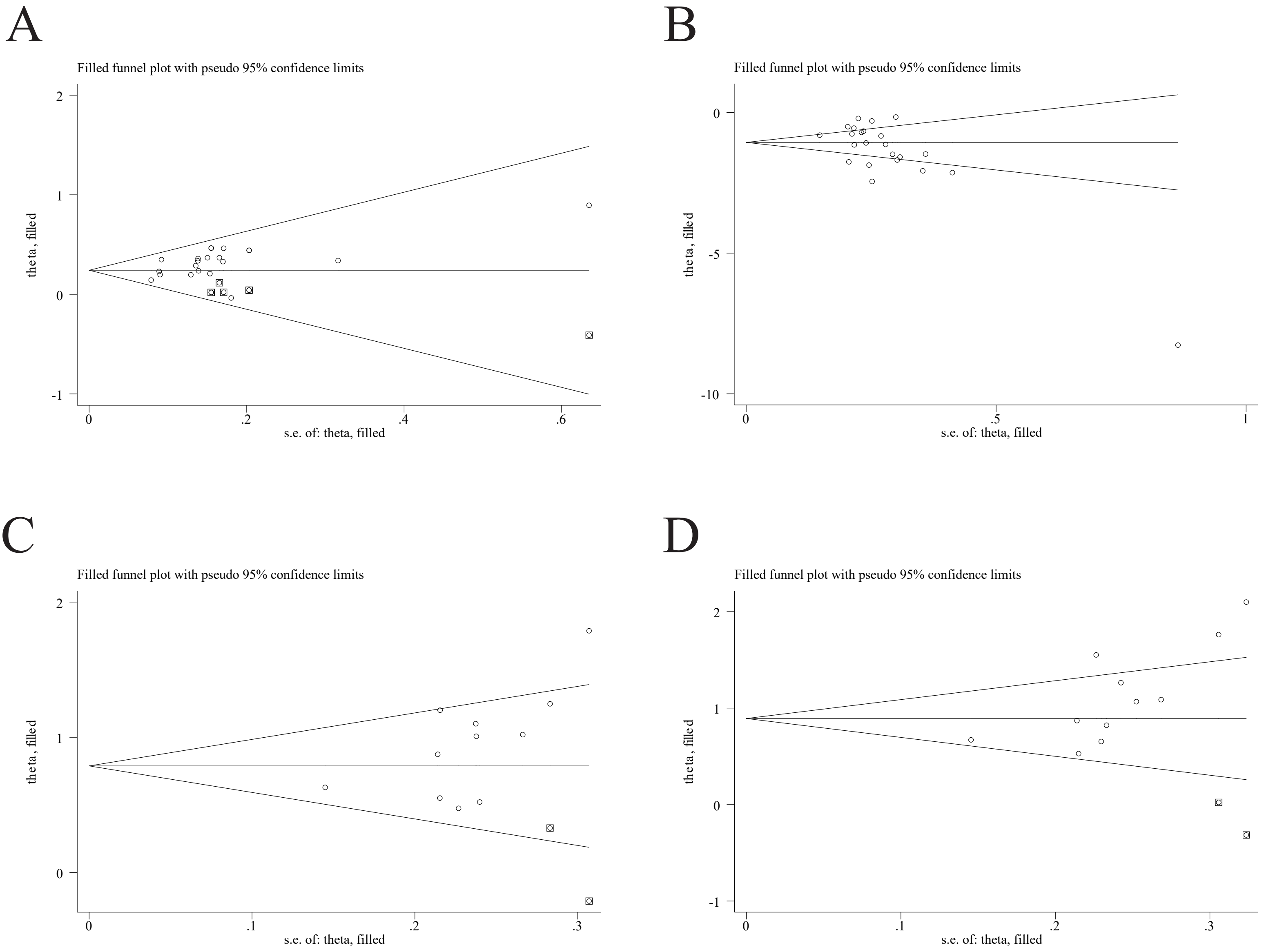

Supplementary Figure 9. Trim and filling method. (A) Trim and filling method of Total effective rate. (B) Trim and filling method of Pain intensity. (C) Trim and filling method of MCV. (D) Trim and filling method of SCV.

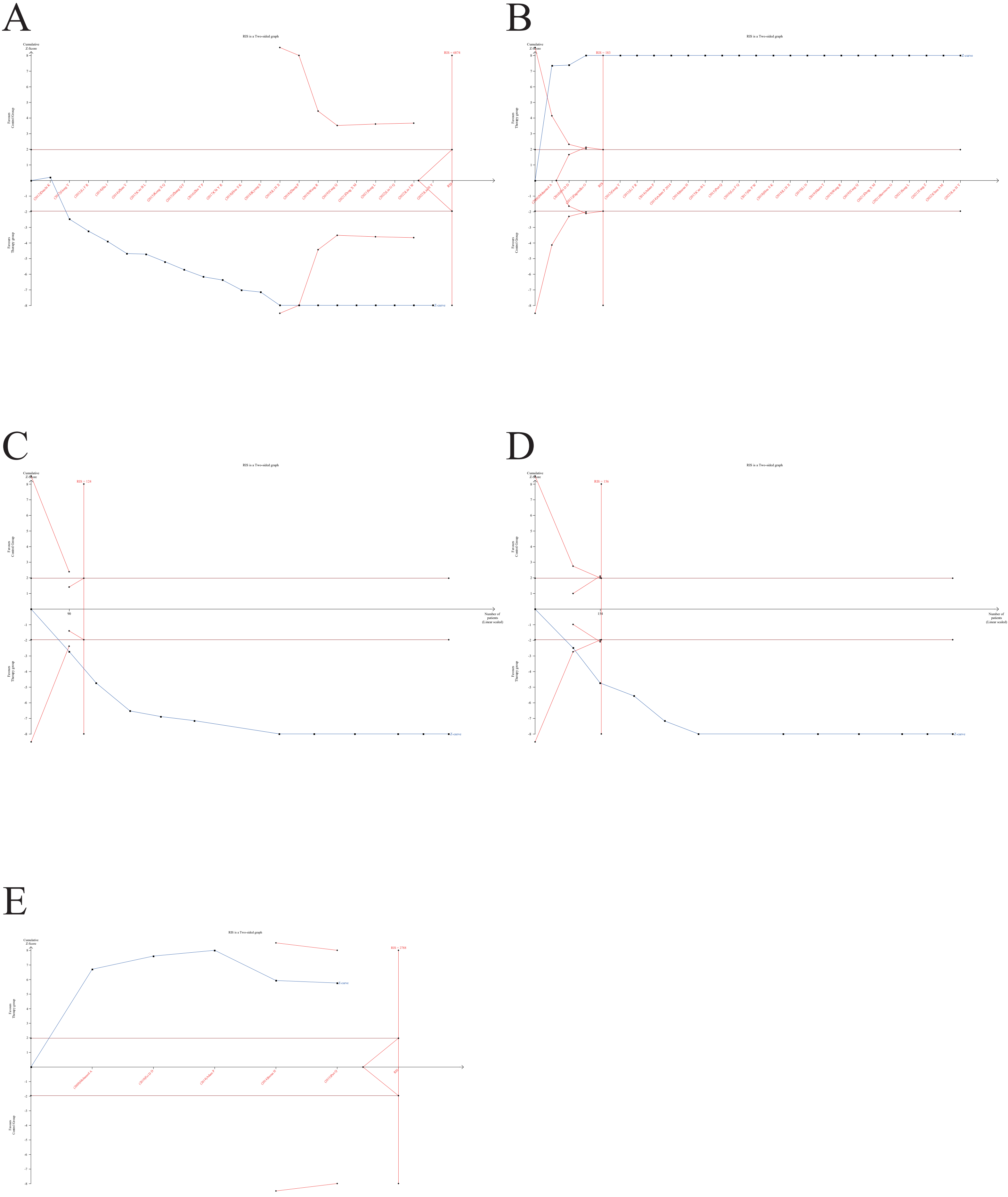

Supplementary Figure 10. Trial sequential analysis (TSA). (A) TSA of Total effective rate. (B) TSA of Pain intensity. (C) TSA of MCV. (D) TSA of SCV. (E) TSA of Depression score.

Supplementary Table 1. The assessment of risk of bias

Adam P 2014

Bias	Authors' judgement	Support for judgement
Random sequence generation (selection bias)	Low risk	Using a random number table
Allocation concealment (selection bias)	Unclear risk	Not mentioned
Blinding of participants and personnel(performance bias)	Unclear risk	Not mentioned
Blinding of outcome assessment (detection bias)	Unclear risk	Not mentioned
Incomplete outcome data (attrition bias)	Unclear risk	Not mentioned
Selective reporting (reporting bias)	High risk	Not all the study's primary outcomes have been reported. Only the pain threshold was reported.
Other bias	Low risk	No other sources of bias

Cao B L 2015

Bias	Authors' judgement	Support for judgement
Random sequence generation (selection bias)	Low risk	Using a random number table
Allocation concealment (selection bias)	Unclear risk	Not mentioned
Blinding of participants and personnel (performance bias)	Unclear risk	Not mentioned
Blinding of outcome assessment (detection bias)	Unclear risk	Not mentioned
Incomplete outcome data (attrition bias)	Low risk	No missing outcome data
Selective reporting (reporting bias)	Unclear risk	All the primary outcomes were reported, but the parts of secondary outcomes were not reported.
Other bias	Low risk	No other sources of bias

Chen S M 2022

Bias	Authors' judgement	Support for judgement
Random sequence generation (selection bias)	Low risk	Using a random number table

Allocation concealment (selection bias)	Unclear risk	Not mentioned
Blinding of participants and personnel (performance bias)	Unclear risk	Not mentioned
Blinding of outcome assessment (detection bias)	Unclear risk	Not mentioned
Incomplete outcome data (attrition bias)	Low risk	No missing outcome data
Selective reporting (reporting bias)	High risk	Not all the study's primary outcomes have been reported. Only the pain threshold was reported.
Other bias	Low risk	No other sources of bias

Chi Y R 2017

**Bias**	**Authors' judgement**	**Support for judgement**
Random sequence generation (selection bias)	Unclear risk	The specific method of randomization which was chosen was not reported
Allocation concealment (selection bias)	Unclear risk	Not mentioned
Blinding of participants and personnel (performance bias)	Unclear risk	Not mentioned
Blinding of outcome assessment (detection bias)	Unclear risk	Not mentioned
Incomplete outcome data (attrition bias)	Low risk	No missing outcome data
Selective reporting (reporting bias)	High risk	Not all the study's primary outcomes have been reported. Only the total effective rate was reported.
Other bias	Low risk	No other sources of bias

Daichi K 2012

**Bias**	**Authors' judgement**	**Support for judgement**
Random sequence generation (selection bias)	Unclear risk	Not mentioned
Allocation concealment (selection bias)	Unclear risk	Not mentioned
Blinding of participants and personnel(performance bias)	Unclear risk	Not mentioned
Blinding of outcome assessment (detection bias)	Unclear risk	Not mentioned
Incomplete outcome data	Unclear risk	Not mentioned

(attrition bias)		
Selective reporting (reporting bias)	High risk	Not all the study's primary outcomes have been reported. Only the total effective was reported.
Other bias	Low risk	No other sources of bias

Deng X M 2021

**Bias**	**Authors' judgement**	**Support for judgement**
Random sequence generation (selection bias)	Low risk	Using a random number table
Allocation concealment (selection bias)	Low risk	Random numbers and group assigned were kept in sealed opaque envelopes.
Blinding of participants and personnel (performance bias)	Unclear risk	Not mentioned
Blinding of outcome assessment (detection bias)	Unclear risk	Not mentioned
Incomplete outcome data (attrition bias)	Low risk	No missing outcome data
Selective reporting (reporting bias)	Unclear risk	All the primary outcomes were reported, but the parts of secondary outcomes were not reported.
Other bias	Low risk	No other sources of bias

Fei D D 2010

**Bias**	**Authors' judgement**	**Support for judgement**
Random sequence generation (selection bias)	Unclear risk	The specific method of randomization which was chosen was not reported.
Allocation concealment (selection bias)	Unclear risk	Not mentioned
Blinding of participants and personnel (performance bias)	Unclear risk	Not mentioned
Blinding of outcome assessment (detection bias)	Unclear risk	Not mentioned
Incomplete outcome data (attrition bias)	Low risk	No missing outcome data
Selective reporting (reporting bias)	High risk	Not all the study's primary outcomes have been reported. Only the pain threshold was reported.
Other bias	Low risk	No other sources of bias

Gong Y 2012

**Bias**	**Authors'**	**Support for judgement**

	judgement	
Random sequence generation (selection bias)	Unclear risk	The specific method of randomization which was chosen was not reported.
Allocation concealment (selection bias)	Unclear risk	Not mentioned
Blinding of participants and personnel (performance bias)	Unclear risk	Not mentioned
Blinding of outcome assessment (detection bias)	Unclear risk	Not mentioned
Incomplete outcome data (attrition bias)	Low risk	No missing outcome data
Selective reporting (reporting bias)	Unclear risk	All the primary outcomes were reported, but the parts of secondary outcomes were not reported.
Other bias	Low risk	No other sources of bias

He P W 2017

**Bias**	**Authors' judgement**	**Support for judgement**
Random sequence generation (selection bias)	Unclear risk	The specific method of randomization which was chosen was not reported.
Allocation concealment (selection bias)	Unclear risk	Not mentioned
Blinding of participants and personnel (performance bias)	Unclear risk	Not mentioned
Blinding of outcome assessment (detection bias)	Unclear risk	Not mentioned
Incomplete outcome data (attrition bias)	Low risk	No missing outcome data
Selective reporting (reporting bias)	High risk	Not all the study's primary outcomes have been reported. Only the pain threshold was reported.
Other bias	Low risk	No other sources of bias

Hou S K 2018

**Bias**	**Authors' judgement**	**Support for judgement**
Random sequence generation (selection bias)	Low risk	Using a random number table
Allocation concealment (selection bias)	Unclear risk	Not mentioned
Blinding of participants and personnel (performance bias)	Unclear risk	Not mentioned

Blinding of outcome assessment (detection bias)	Unclear risk	Not mentioned
Incomplete outcome data (attrition bias)	Low risk	No missing outcome data
Selective reporting (reporting bias)	Unclear risk	All the primary outcomes were reported, but the parts of secondary outcomes were not reported.
Other bias	Low risk	No other sources of bias

Hu J 2014

Bias	Authors' judgement	Support for judgement
Random sequence generation (selection bias)	Low risk	Using a random number table
Allocation concealment (selection bias)	Unclear risk	Not mentioned
Blinding of participants and personnel (performance bias)	Unclear risk	Not mentioned
Blinding of outcome assessment (detection bias)	Unclear risk	Not mentioned
Incomplete outcome data (attrition bias)	Low risk	No missing outcome data
Selective reporting (reporting bias)	High risk	Not all the study's primary outcomes have been reported. Only the total effective was reported.
Other bias	Low risk	No other sources of bias

Imran H 2014

Bias	Authors' judgement	Support for judgement
Random sequence generation (selection bias)	High risk	The non-probabilistic purpose sampling technique is adopted.
Allocation concealment (selection bias)	Unclear risk	Not mentioned
Blinding of participants and personnel (performance bias)	Unclear risk	Not mentioned
Blinding of outcome assessment (detection bias)	Unclear risk	Not mentioned
Incomplete outcome data (attrition bias)	Unclear risk	Not mentioned
Selective reporting (reporting bias)	High risk	Not all the study's primary outcomes have been reported. Only the pain threshold was reported.
Other bias	Low risk	No other sources of bias

Kong X Q 2015

Bias	Authors' judgement	Support for judgement
Random sequence generation (selection bias)	Unclear risk	The specific method of randomization which was chosen was not reported.
Allocation concealment (selection bias)	Unclear risk	Not mentioned
Blinding of participants and personnel (performance bias)	Unclear risk	Not mentioned
Blinding of outcome assessment (detection bias)	Unclear risk	Not mentioned
Incomplete outcome data (attrition bias)	Low risk	No missing outcome data
Selective reporting (reporting bias)	High risk	Not all the study's primary outcomes have been reported. Only the total effective was reported.
Other bias	Low risk	No other sources of bias

Kyung S 2018

Bias	Authors' judgement	Support for judgement
Random sequence generation (selection bias)	Low risk	Using a random number table
Allocation concealment (selection bias)	Low risk	Random numbers and group assigned were kept in sealed opaque envelopes.
Blinding of participants and personnel (performance bias)	Unclear risk	Not mentioned
Blinding of outcome assessment (detection bias)	Unclear risk	Not mentioned
Incomplete outcome data (attrition bias)	Low risk	No missing outcome data
Selective reporting (reporting bias)	High risk	Not all the study's primary outcomes have been reported. Only the total effective was reported.
Other bias	Low risk	No other sources of bias

Li F R 2013

Bias	Authors' judgement	Support for judgement
Random sequence generation (selection bias)	Low risk	Using a random number table
Allocation concealment (selection bias)	Unclear risk	Not mentioned
Blinding of participants and	Unclear risk	Not mentioned

personnel (performance bias)		
Blinding of outcome assessment (detection bias)	Unclear risk	Not mentioned
Incomplete outcome data (attrition bias)	Low risk	No missing outcome data
Selective reporting (reporting bias)	Unclear risk	All the primary outcomes were reported, but the parts of secondary outcomes were not reported.
Other bias	Low risk	No other sources of bias

Li H X 2018

**Bias**	**Authors' judgement**	**Support for judgement**
Random sequence generation (selection bias)	Low risk	Using a random number table
Allocation concealment (selection bias)	Unclear risk	Not mentioned
Blinding of participants and personnel (performance bias)	Unclear risk	Not mentioned
Blinding of outcome assessment (detection bias)	Unclear risk	Not mentioned
Incomplete outcome data (attrition bias)	Low risk	No missing outcome data
Selective reporting (reporting bias)	Unclear risk	All the primary outcomes were reported, but the parts of secondary outcomes were not reported.
Other bias	Low risk	No other sources of bias

Li N 2019

**Bias**	**Authors' judgement**	**Support for judgement**
Random sequence generation (selection bias)	Unclear risk	Not mentioned
Allocation concealment (selection bias)	Unclear risk	Not mentioned
Blinding of participants and personnel (performance bias)	Unclear risk	Not mentioned
Blinding of outcome assessment (detection bias)	Unclear risk	Not mentioned
Incomplete outcome data (attrition bias)	Low risk	No missing outcome data
Selective reporting (reporting bias)	High risk	Not all the study's primary outcomes have been reported. Only the pain threshold was reported.

Other bias	Low risk	No other sources of bias

Liu F Q 2016

Bias	Authors' judgement	Support for judgement
Random sequence generation (selection bias)	Low risk	Using a random number table
Allocation concealment (selection bias)	Unclear risk	Not mentioned
Blinding of participants and personnel (performance bias)	Unclear risk	Not mentioned
Blinding of outcome assessment (detection bias)	Unclear risk	Not mentioned
Incomplete outcome data (attrition bias)	Low risk	No missing outcome data
Selective reporting (reporting bias)	High risk	Not all the study's primary outcomes have been reported. Only the pain threshold was reported.
Other bias	Low risk	No other sources of bias

Liu G Q 2022

Bias	Authors' judgement	Support for judgement
Random sequence generation (selection bias)	Unclear risk	Not mentioned
Allocation concealment (selection bias)	Unclear risk	Not mentioned
Blinding of participants and personnel (performance bias)	Unclear risk	Not mentioned
Blinding of outcome assessment (detection bias)	Unclear risk	Not mentioned
Incomplete outcome data (attrition bias)	Low risk	No missing outcome data
Selective reporting (reporting bias)	High risk	Not all the study's primary outcomes have been reported. Only the total effective was reported.
Other bias	Low risk	No other sources of bias

Liu H Y 2023

Bias	Authors' judgement	Support for judgement
Random sequence generation (selection bias)	Low risk	Using a random number table

Allocation concealment (selection bias)	Unclear risk	Not mentioned
Blinding of participants and personnel (performance bias)	Unclear risk	Not mentioned
Blinding of outcome assessment (detection bias)	Unclear risk	Not mentioned
Incomplete outcome data (attrition bias)	Low risk	No missing outcome data
Selective reporting (reporting bias)	Unclear risk	All the primary outcomes were reported, but the parts of secondary outcomes were not reported.
Other bias	Low risk	No other sources of bias

Liu J W 2022

**Bias**	**Authors' judgement**	**Support for judgement**
Random sequence generation (selection bias)	Low risk	Using a random number table
Allocation concealment (selection bias)	Unclear risk	Not mentioned
Blinding of participants and personnel (performance bias)	Unclear risk	Not mentioned
Blinding of outcome assessment (detection bias)	Unclear risk	Not mentioned
Incomplete outcome data (attrition bias)	Low risk	No missing outcome data
Selective reporting (reporting bias)	High risk	Not all the study's primary outcomes have been reported. Only the total effective was reported.
Other bias	Low risk	No other sources of bias

Maria T 2019

**Bias**	**Authors' judgement**	**Support for judgement**
Random sequence generation (selection bias)	Low risk	Using a computerized group assignment
Allocation concealment (selection bias)	Low risk	The study data manager is unaware of the participant assignment
Blinding of participants and personnel (performance bias)	Unclear risk	Not mentioned
Blinding of outcome assessment (detection bias)	Low risk	Outcome assessors and statisticians were blinded to intervention assignment.
Incomplete outcome data	Low risk	No missing outcome data

(attrition bias)		
Selective reporting (reporting bias)	High risk	Not all the study's primary outcomes have been reported. Only the pain threshold was reported.
Other bias	Low risk	No other sources of bias

Mohamed A 2000

Bias	Authors' judgement	Support for judgement
Random sequence generation (selection bias)	Unclear risk	Not mentioned
Allocation concealment (selection bias)	Unclear risk	Not mentioned
Blinding of participants and personnel (performance bias)	Low risk	The subjects were given false electrical stimulation
Blinding of outcome assessment (detection bias)	Low risk	Outcome assessors and statisticians were blinded to intervention assignment.
Incomplete outcome data (attrition bias)	Low risk	No missing outcome data
Selective reporting (reporting bias)	High risk	Not all the study's primary outcomes have been reported. Only the pain threshold was reported.
Other bias	Low risk	No other sources of bias

Pan Q 2015

Bias	Authors' judgement	Support for judgement
Random sequence generation (selection bias)	Low risk	Using a random number table
Allocation concealment (selection bias)	Unclear risk	Not mentioned
Blinding of participants and personnel (performance bias)	Unclear risk	Not mentioned
Blinding of outcome assessment (detection bias)	Unclear risk	Not mentioned
Incomplete outcome data (attrition bias)	Low risk	No missing outcome data
Selective reporting (reporting bias)	High risk	Not all the study's primary outcomes have been reported. Only the pain threshold was reported.
Other bias	Low risk	No other sources of bias

Shevtsova G 2021

Bias	Authors'	Support for judgement

	judgement	
Random sequence generation (selection bias)	Low risk	Using the sealed envelope method
Allocation concealment (selection bias)	Low risk	Random numbers and group assigned were kept in sealed opaque envelopes.
Blinding of participants and personnel (performance bias)	Unclear risk	Not mentioned
Blinding of outcome assessment (detection bias)	Unclear risk	Not mentioned
Incomplete outcome data (attrition bias)	Unclear risk	Not mentioned
Selective reporting (reporting bias)	High risk	Not all the study's primary outcomes have been reported. Only the pain threshold was reported.
Other bias	Low risk	No other sources of bias

Song L 2021

**Bias**	**Authors' judgement**	**Support for judgement**
Random sequence generation (selection bias)	Low risk	Using stratified blocked randomization
Allocation concealment (selection bias)	Unclear risk	Not mentioned
Blinding of participants and personnel (performance bias)	Unclear risk	Not mentioned
Blinding of outcome assessment (detection bias)	Unclear risk	Not mentioned
Incomplete outcome data (attrition bias)	Low risk	No missing outcome data
Selective reporting (reporting bias)	Unclear risk	All the primary outcomes were reported, but the parts of secondary outcomes were not reported.
Other bias	Low risk	No other sources of bias

Wang R 2019

**Bias**	**Authors' judgement**	**Support for judgement**
Random sequence generation (selection bias)	Unclear risk	Not mentioned
Allocation concealment (selection bias)	Unclear risk	Not mentioned
Blinding of participants and personnel (performance bias)	Unclear risk	Not mentioned

Blinding of outcome assessment (detection bias)	Unclear risk	Not mentioned
Incomplete outcome data (attrition bias)	Unclear risk	Not mentioned
Selective reporting (reporting bias)	Unclear risk	All the primary outcomes were reported, but the parts of secondary outcomes were not reported.
Other bias	Low risk	No other sources of bias

Yang D L 2017

**Bias**	**Authors' judgement**	**Support for judgement**
Random sequence generation (selection bias)	Unclear risk	Not mentioned
Allocation concealment (selection bias)	Unclear risk	Not mentioned
Blinding of participants and personnel (performance bias)	Unclear risk	Not mentioned
Blinding of outcome assessment (detection bias)	Unclear risk	Not mentioned
Incomplete outcome data (attrition bias)	Low risk	No missing outcome data
Selective reporting (reporting bias)	High risk	Not all the study's primary outcomes have been reported. Only the pain threshold was reported.
Other bias	Low risk	No other sources of bias

Yang Q 2019

**Bias**	**Authors' judgement**	**Support for judgement**
Random sequence generation (selection bias)	Unclear risk	Not mentioned
Allocation concealment (selection bias)	Unclear risk	Not mentioned
Blinding of participants and personnel (performance bias)	Unclear risk	Not mentioned
Blinding of outcome assessment (detection bias)	Unclear risk	Not mentioned
Incomplete outcome data (attrition bias)	Low risk	No missing outcome data
Selective reporting (reporting bias)	Unclear risk	All the primary outcomes were reported, but the parts of secondary outcomes were not reported.
Other bias	Low risk	No other sources of bias

Yang Z 2021

Bias	Authors' judgement	Support for judgement
Random sequence generation (selection bias)	Low risk	Using a random number table
Allocation concealment (selection bias)	Unclear risk	Not mentioned
Blinding of participants and personnel (performance bias)	Unclear risk	Not mentioned
Blinding of outcome assessment (detection bias)	Unclear risk	Not mentioned
Incomplete outcome data (attrition bias)	Low risk	No missing outcome data
Selective reporting (reporting bias)	High risk	Not all the study's primary outcomes have been reported. Only the pain threshold was reported.
Other bias	Low risk	No other sources of bias

Zagorulko O 2011

Bias	Authors' judgement	Support for judgement
Random sequence generation (selection bias)	Unclear risk	Not mentioned
Allocation concealment (selection bias)	Unclear risk	Not mentioned
Blinding of participants and personnel (performance bias)	Unclear risk	Not mentioned
Blinding of outcome assessment (detection bias)	Unclear risk	Not mentioned
Incomplete outcome data (attrition bias)	Unclear risk	Not mentioned
Selective reporting (reporting bias)	High risk	Not all the study's primary outcomes have been reported. Only the pain threshold was reported.
Other bias	Low risk	No other sources of bias

Zhang P 2018

Bias	Authors' judgement	Support for judgement
Random sequence generation (selection bias)	High risk	Grouped according to the order of visit
Allocation concealment (selection bias)	Unclear risk	Not mentioned
Blinding of participants and	Unclear risk	Not mentioned

personnel (performance bias)		
Blinding of outcome assessment (detection bias)	Unclear risk	Not mentioned
Incomplete outcome data (attrition bias)	Low risk	No missing outcome data
Selective reporting (reporting bias)	High risk	Not all the study's primary outcomes have been reported. Only the total effective was reported.
Other bias	Low risk	No other sources of bias

Zhang P X 2019

**Bias**	**Authors' judgement**	**Support for judgement**
Random sequence generation (selection bias)	Low risk	Using a random number table
Allocation concealment (selection bias)	Unclear risk	Not mentioned
Blinding of participants and personnel (performance bias)	Unclear risk	Not mentioned
Blinding of outcome assessment (detection bias)	Unclear risk	Not mentioned
Incomplete outcome data (attrition bias)	Low risk	No missing outcome data
Selective reporting (reporting bias)	High risk	Not all the study's primary outcomes have been reported.
Other bias	Low risk	No other sources of bias

Zhang Q F 2015

**Bias**	**Authors' judgement**	**Support for judgement**
Random sequence generation (selection bias)	Unclear risk	Not mentioned
Allocation concealment (selection bias)	Unclear risk	Not mentioned
Blinding of participants and personnel (performance bias)	Unclear risk	Not mentioned
Blinding of outcome assessment (detection bias)	Unclear risk	Not mentioned
Incomplete outcome data (attrition bias)	Low risk	No missing outcome data
Selective reporting (reporting bias)	High risk	Not all the study's primary outcomes have been reported. Only the total effective was reported.

Other bias	Low risk	No other sources of bias

Zhan Y 2014

**Bias**	**Authors' judgement**	**Support for judgement**
Random sequence generation (selection bias)	Unclear risk	Not mentioned
Allocation concealment (selection bias)	Unclear risk	Not mentioned
Blinding of participants and personnel (performance bias)	Unclear risk	Not mentioned
Blinding of outcome assessment (detection bias)	Unclear risk	Not mentioned
Incomplete outcome data (attrition bias)	Low risk	No missing outcome data
Selective reporting (reporting bias)	High risk	Not all the study's primary outcomes have been reported. Only the total effective was reported.
Other bias	Low risk	No other sources of bias

Zhu Y P 2016

**Bias**	**Authors' judgement**	**Support for judgement**
Random sequence generation (selection bias)	High risk	Grouped according to the order of visit
Allocation concealment (selection bias)	Unclear risk	Not mentioned
Blinding of participants and personnel (performance bias)	Unclear risk	Not mentioned
Blinding of outcome assessment (detection bias)	Unclear risk	Not mentioned
Incomplete outcome data (attrition bias)	Low risk	No missing outcome data
Selective reporting (reporting bias)	High risk	Not all the study's primary outcomes have been reported. Only the total effective was reported.
Other bias	Low risk	No other sources of bias

Supplementary Table 2. Sensitivity analyses for outcomes.

Total effective rate

Excluded study	Intervention group(n)	Control group(n)	RR/SMD (95% CI)	P value	Heterogeneity test	Effect model
Before excluding	821	802	1.42 [1.34, 1.52]	<0.001	P = 0.08, I^2 = 32%	Fixed
Cao B L 2015	790	733	1.43 [1.34, 1.52]	<0.001	P = 0.06, I^2 = 36%	Fixed
Chi Y R 2017	801	782	1.43 [1.34, 1.53]	<0.001	P = 0.06, I^2 = 35%	Fixed
Daichi K 2012	801	784	1.44 [1.35, 1.54]	<0.001	P = 0.12, I^2 = 28%	Fixed
Deng X M 2021	791	772	1.37 [1.28, 1.46]	<0.001	P = 0.57, I^2 = 0%	Fixed
Gong Y 2012	781	762	1.42 [1.33, 1.51]	<0.001	P = 0.09, I^2 = 32%	Fixed
Hou S K 2018	781	762	1.42 [1.33, 1.51]	<0.001	P = 0.09, I^2 = 32%	Fixed
Hu J 2014	781	762	1.42 [1.33, 1.52]	<0.001	P = 0.07, I^2 = 33%	Fixed
Kong X Q 2015	781	762	1.42 [1.33, 1.52]	<0.001	P = 0.07, I^2 = 33%	Fixed
Kyung S 2018	767	758	1.42 [1.33, 1.51]	<0.001	P = 0.09, I^2 = 32%	Fixed
Li F R 2013	771	752	1.43 [1.34, 1.53]	<0.001	P = 0.05, I^2 = 37%	Fixed
Li H X 2018	721	702	1.43 [1.33, 1.53]	<0.001	P = 0.06, I^2 = 36%	Fixed
Liu G Q 2022	791	772	1.43 [1.33, 1.52]	<0.001	P = 0.06, I^2 = 36%	Fixed
Liu H Y 2023	794	776	1.43 [1.34, 1.53]	<0.001	P = 0.06, I^2 = 35%	Fixed
Liu J W 2022	791	772	1.43 [1.34, 1.52]	<0.001	P = 0.06, I^2 = 36%	Fixed
Song L 2021	789	770	1.44 [1.34, 1.54]	<0.001	P = 0.06, I^2 = 35%	Fixed
Wang R 2019	771	756	1.42 [1.33, 1.52]	<0.001	P = 0.06, I^2 = 35%	Fixed
Yang Q 2019	773	754	1.45 [1.35, 1.55]	<0.001	P = 0.18, I^2 = 22%	Fixed
Zhang P 2018	780	761	1.44 [1.35, 1.54]	<0.001	P = 0.08, I^2 = 32%	Fixed
Zhang Q F 2015	781	762	1.44 [1.34, 1.54]	<0.001	P = 0.07, I^2 = 34%	Fixed
Zhan Y 2014	791	772	1.42 [1.33, 1.52]	<0.001	P = 0.08, I^2 = 33%	Fixed
Zhu Y P 2016	793	774	1.42 [1.33, 1.52]	<0.001	P = 0.06, I^2 = 35%	Fixed

Pain intensity

Excluded study	Intervention group(n)	Control group(n)	RR/SMD (95% CI)	P value	Heterogeneity test	Effect model
Before excluding	933	898	-1.27 [-1.58, -0.95]	<0.001	P <0.001, I^2 =89%	Random
Adam P 2014	909	877	-1.32 [-1.63, -1.00]	<0.001	P <0.001, I^2 =89%	Random
Cao B L 2015	902	869	-1.29 [-1.62, -0.97]	<0.001	P <0.001, I^2 =89%	Random
Chen S M 2022	903	868	-1.25 [-1.57, -0.93]	<0.001	P <0.001, I^2 =89%	Random
Deng X M 2021	903	868	-1.26 [-1.58, -0.94]	<0.001	P <0.001, I^2 =89%	Random
Fei D D 2010	885	856	-1.30 [-1.63, -0.98]	<0.001	P <0.001, I^2 =89%	Random

Gong Y 2012	893	858	-1.30 [-1.62, -0.97]	<0.001	P <0.001, I² =89%	Random
He P W 2017	893	858	-1.28 [-1.61, -0.95]	<0.001	P <0.001, I² =89%	Random
Hou S K 2018	893	858	-1.32 [-1.63, -1.00]	<0.001	P <0.001, I² =89%	Random
Imran H 2014	871	848	-1.20 [-1.50, -0.90]	<0.001	P <0.001, I² =87%	Random
Li F R 2013	883	848	-1.31 [-1.63, -0.98]	<0.001	P <0.001, I² =89%	Random
Li H X 2018	833	798	-1.30 [-1.64, -0.96]	<0.001	P <0.001, I² =89%	Random
Li N 2019	833	848	-1.28 [-1.61, -0.95]	<0.001	P <0.001, I² =89%	Random
Liu F Q 2016	905	870	-1.26 [-1.58, -0.93]	<0.001	P <0.001, I² =89%	Random
Liu H Y 2023	906	872	-1.12 [-1.38, -0.86]	<0.001	P <0.001, I² =84%	Random
Maria T 2019	907	884	-1.23 [-1.55, -0.92]	<0.001	P <0.001, I² =89%	Random
Mohamed A 2000	907	873	-1.23 [-1.55, -0.92]	<0.001	P <0.001, I² =89%	Random
Pan Q 2015	897	857	-1.30 [-1.63, -0.97]	<0.001	P <0.001, I² =89%	Random
Shevtsova G 2021	867	832	-1.24 [-1.56, -0.92]	<0.001	P <0.001, I² =89%	Random
Song L 2021	901	866	-1.31 [-1.63, -0.99]	<0.001	P <0.001, I² =89%	Random
Wang R 2019	833	852	-1.24 [-1.56, -0.92]	<0.001	P <0.001, I² =89%	Random
Yang Q 2019	885	850	-1.30 [-1.62, -0.97]	<0.001	P <0.001, I² =89%	Random
Yang Z 2021	903	868	-1.28 [-1.60, -0.95]	<0.001	P <0.001, I² =89%	Random
Zagorulko O 2011	913	878	-1.26 [-1.58, -0.94]	<0.001	P <0.001, I² =89%	Random

MCV

Excluded study	Intervention group(n)	Control group(n)	RR/SMD (95% CI)	P value	Heterogeneity test	Effect model
Before excluding	498	488	3.58 [2.77, 4.38]	<0.001	P = 0.002, I² = 63%	Random
Deng X M 2021	468	458	3.58 [2.77, 4.38]	<0.001	P = 0.006, I² =61%	Random
Fei D D 2010	450	446	3.58 [2.77, 4.38]	<0.001	P = 0.002, I² = 65%	Random
He P W 2017	458	448	3.59 [2.70, 4.48]	<0.001	P = 0.001, I² = 67%	Random
Hou S K 2018	458	448	3.69 [2.83, 4.55]	<0.001	P = 0.002, I² = 66%	Random
Li H X 2018	398	388	3.73 [2.84, 4.63]	<0.001	P = 0.003, I² = 64%	Random
Liu J W 2022	468	458	3.07 [2.60, 3.54]	<0.001	P = 0.008, I² = 41%	Fixed
Wang R 2019	448	442	3.41 [2.63, 4.19]	<0.001	P = 0.007, I² = 60%	Random
Yang D L 2017	462	452	3.58 [2.73, 4.42]	<0.001	P = 0.001, I² = 67%	Random
Zhang P 2018	457	447	3.68 [2.76, 4.61]	<0.001	P = 0.002, I² = 66%	Random
Zhang P X 2019	447	437	3.43 [2.60, 4.25]	<0.001	P = 0.006, I² =61%	Random
Zhan Y 2014	466	456	3.55 [2.68, 4.42]	<0.001	P = 0.002, I² = 66%	Random

SCV

Excluded study	Intervention group(n)	Control group(n)	RR/SMD (95% CI)	P value	Heterogeneity test	Effect model
Before excluding	498	488	3.62 [2.75, 4.49]	<0.001	P <0.001, I² = 78%	Random
Deng X M 2021	468	458	3.75 [2.73, 4.77]	<0.001	P <0.001, I² =80%	Random
Fei D D 2010	450	446	3.80 [2.88, 4.73]	<0.001	P <0.001, I² = 79%	Random
He P W 2017	458	448	3.69 [2.75, 4.62]	<0.001	P <0.001, I² =80%	Random
Hou S K 2018	458	448	3.66 [2.72, 4.60]	<0.001	P <0.001, I² =80%	Random
Li H X 2018	398	388	3.72 [2.75, 4.69]	<0.001	P <0.001, I² =80%	Random
Liu J W 2022	468	458	2.96 [2.58, 3.34]	<0.001	P = 0.04, I² = 48%	Fixed
Wang R 2019	448	442	3.42 [2.59, 4.26]	<0.001	P <0.001, I² =77%	Random
Yang D L 2017	462	452	3.70 [2.74, 4.67]	<0.001	P <0.001, I² =80%	Random
Zhang P 2018	457	447	3.82 [2.87, 4.77]	<0.001	P <0.001, I² =76%	Random
Zhang P X 2019	447	437	3.64 [2.65, 4.62]	<0.001	P <0.001, I² =79%	Random
Zhan Y 2014	466	456	3.62 [2.68, 4.56]	<0.001	P <0.001, I² =80%	Random

Depression score

Excluded study	Intervention group(n)	Control group(n)	RR/SMD (95% CI)	P value	Heterogeneity test	Effect model
Before excluding	195	179	-1.02 [-1.58, -0.46]	<0.001	P <0.001, I² = 84%	Random
Adam P 2014	171	158	-0.82 [-1.35, -0.30]	0.002	P = 0.002, I² = 80%	Random
Fei D D 2010	147	137	-1.11 [-1.87, -0.35]	0.004	P <0.001, I² = 88%	Random
Imran H 2014	133	129	-1.17 [-1.91, -0.44]	0.002	P <0.001, I² = 86%	Random
Mohamed A 2000	170	154	-0.82 [-1.34, -0.30]	0.002	P = 0.002, I² = 79%	Random
Pan Q 2015	159	138	-1.21 [-1.87, -0.56]	<0.001	P <0.001, I² = 84%	Random

TCSS

Excluded study	Intervention group(n)	Control group(n)	RR/SMD (95% CI)	P value	Heterogeneity test	Effect model
Before excluding	147	142	-1.27 [-1.58, -0.95]	<0.001	P = 0.005, I² = 76%	Random
Chen S M 2022	117	112	-2.16 [-3.49, -0.83]	0.001	P = 0.01, I² = 78%	Random
He P W 2017	107	102	-2.06 [-3.20, -0.92]	<0.001	P = 0.009, I² = 79%	Random
Liu H Y 2023	120	116	-2.71 [-3.72, -1.69]	<0.001	P = 0.02, I² = 74%	Random

Wang R 2019	97	96	-2.69 [-3.74, -1.63]	<0.001	P = 0.02, I^2 = 74%	Random

QOL

Excluded study	Intervention group(n)	Control group(n)	RR/SMD (95% CI)	P value	Heterogeneity test	Effect model
Before excluding	100	97	1.83 [-1.90, 5.57]	0.34	P <0.001, I^2 = 99%	Random
Adam P 2014	76	76	1.87 [-4.74, 8.47]	0.58	P <0.001, I^2 = 99%	Random
Liu F Q 2016	72	69	3.50 [0.11, 6.89]	0.04	P <0.001, I^2 = 97%	Random
Yang Q 2019	52	49	0.14 [-3.07, 3.35]	0.93	P <0.001, I^2 = 98%	Random

TCM syndrome score

Excluded study	Intervention group(n)	Control group(n)	RR/MD (95% CI)	P value	Heterogeneity test	Effect model
Before excluding	97	96	-4.99 [-6.79, -3.18]	<0.001	P = 0.004, I^2 = 82%	Random
He P W 2017	57	56	-4.32 [-6.90, -1.74]	0.001	P = 0.02, I^2 = 81%	Random
Liu H Y 2023	70	70	-5.98 [-6.77, -5.19]	<0.001	P = 0.59, I^2 = 0%	Fixed
Liu J W 2022	67	66	-4.62 [-7.68, -1.57]	0.003	P = 0.001, I^2 = 91%	Random

Adverse events

Excluded study	Intervention group(n)	Control group(n)	RR/SMD (95% CI)	P value	Heterogeneity test	Effect model
Before excluding	170	154	0.88 [0.09, 8.24]	0.91	P = 0.006, I^2 = 76%	Random
Fei D D 2010	122	112	0.66 [0.04, 12.11]	0.78	P = 0.003, I^2 = 83%	Random
Gong Y 2012	130	114	0.38 [0.02, 7.23]	0.52	P = 0.004, I^2 = 82%	Random
Kyung S 2018	116	110	0.95 [0.02, 55.27]	0.98	P = 0.003, I^2 = 83%	Random
Liu F Q 2016	142	126	2.29 [0.30, 17.41]	0.42	P = 0.007, I^2 = 62%	Random

Supplementary Table 3. Egger's test to assess publication bias in the included studies

Std_Eff	Coef.	Std. Err.	t	P>|t|	[95% Conf. Interval]	
Total effective rate	5.185692	1.480059	3.50	0.003	2.076203	8.295181
Pain intensity	-6.701009	1.972531	-3.40	0.003	-10.80311	-2.598906
MCV	4.549962	1.95836	2.32	0.045	0.1198444	8.980079
SCV	6.241927	2.003383	3.12	0.012	1.709961	10.77389
Depression score	-8.84719	3.863033	-2.29	0.149	-25.46848	7.774098
TCSS	-4.102326	11.51285	-0.36	0.782	-150.387	142.1824
Quality of life	52.19084	5.616879	9.29	0.068	-19.17837	123.5601
TCM syndrome score	-60.50669	10.44747	-5.79	0.109	-193.2543	72.24096
Adverse events	0.1140906	1.802431	0.06	0.955	-7.641144	7.869325

Supplementary Table 4. Trim and filling method.

Outcome	Method	Pooled	95% CI		Asymptotic		No. of
		Est	Lower	Upper	z_value	p_value	studies
Total effective rate	Fixed	1.273	1.208	1.343	8.955	0.000	28
Pain intensity	Random	0.277	0.202	0.379	-7.963	0.000	23
MCV	Random	2.241	1.778	2.826	6.826	0.000	13
SCV	Random	2.525	1.874	3.404	6.082	0.000	13
